# Structure-Based Statistical Mechanical Model Accounts for the Causality and Energetics of Allosteric Communication

**DOI:** 10.1371/journal.pcbi.1004678

**Published:** 2016-03-03

**Authors:** Enrico Guarnera, Igor N. Berezovsky

**Affiliations:** 1 Bioinformatics Institute (BII), Agency for Science, Technology and Research (ASTAR), Singapore; 2 Department of Biological Sciences (DBS), National University of Singapore (NUS), Singapore; University of North Texas System College of Pharmacy, UNITED STATES

## Abstract

Allostery is one of the pervasive mechanisms through which proteins in living systems carry out enzymatic activity, cell signaling, and metabolism control. Effective modeling of the protein function regulation requires a synthesis of the thermodynamic and structural views of allostery. We present here a structure-based statistical mechanical model of allostery, allowing one to observe causality of communication between regulatory and functional sites, and to estimate per residue free energy changes. Based on the consideration of ligand free and ligand bound systems in the context of a harmonic model, corresponding sets of characteristic normal modes are obtained and used as inputs for an allosteric potential. This potential quantifies the mean work exerted on a residue due to the local motion of its neighbors. Subsequently, in a statistical mechanical framework the entropic contribution to allosteric free energy of a residue is directly calculated from the comparison of conformational ensembles in the ligand free and ligand bound systems. As a result, this method provides a systematic approach for analyzing the energetics of allosteric communication based on a single structure. The feasibility of the approach was tested on a variety of allosteric proteins, heterogeneous in terms of size, topology and degree of oligomerization. The allosteric free energy calculations show the diversity of ways and complexity of scenarios existing in the phenomenology of allosteric causality and communication. The presented model is a step forward in developing the computational techniques aimed at detecting allosteric sites and obtaining the discriminative power between agonistic and antagonistic effectors, which are among the major goals in allosteric drug design.

## Author Summary

The 50th anniversary of Monod-Changeux-Jacob seminal paper “Allosteric proteins and cellular control systems” became the hallmark of a new wave in the allostery studies and the turning point in our vision of allostery and its implications in protein engineering and drug design. Recent experimental and theoretical works clearly show relevance of allosteric phenomenon to drug design, unraveling advantages of allosteric drugs in comparison to traditional orthosteric compounds. Remarkable simplicity of allosteric effectors and, at the same time, their potentially high specificity is one of the most important traits. The non conserved nature of allosteric ligands is a basis for avoiding drug resistance, and existence of latent regulatory sites make them attractive drug targets. The model presented in this work provides a theoretical framework for the quantification of the causality and energetics of allosteric regulation, which is a prerequisite for design of effector molecules with required characteristics. The synthesis between the thermodynamics of allostery and the intrinsic atomic nature of proteins and their interactions with the allosteric effectors accomplished in this work is a small initial step in the long endeavor towards future allosteric drugs.

## Introduction

Activity modulation through ligand binding in sites other than the catalytic ones is a formidable way of performing biological function in allosteric proteins. Consideration of the experimental activity curves [1] together with the first available hemoglobin X-ray structure [2] had resulted in the introduction of the first theoretical concepts of allostery in the early 1960s. According to the Monod-Wyman-Changeux (MWC) phenomenological model a ligand binding to a symmetric oligomeric protein can shift the equilibrium between active and inactive conformational states, while preserving the symmetry of the oligomer [3]. The cornerstone of Koshland-Nemethy-Filmer (KNM) model is the “induced-fit” mechanism, where ligand binding to one of the monomers triggers conformational changes in its tertiary structure, which, in turn, lead to rearrangements in other parts of the oligomer with no structural symmetry preserved [4]. Despite their conceptual importance, MWC and KNF models share common limitations, such as the requirement of considering only oligomeric structures, and the lack of atomic level description. Additionally, the difference in the kinetics of the process—ligand binding precedes conformational change (KNF) or the opposite (MWC)—is a source of dichotomy between these phenomenological models.

Frauenfelder’s concept of the energy landscape with multiple conformational states [5–9] complemented by the development of NMR methods [10] have motivated a thermodynamic view of allostery as the foundation for the atomic level description [11]. Based on the statistical physics of protein dynamics, Cooper and Dryden showed that binding of the allosteric effector can induce changes in localization and frequency of fluctuations, which may result in cooperative binding even in the absence of substantial conformational changes [12]. Numerous experimental evidences [13, 11, 14], X-ray crystallography of thousands of proteins showed that allosteric regulation is omnipresent, and it should be studied in all types of proteins, including both oligomers and monomers, small single-domain enzymes and huge molecular machines [15, 16], receptors [17, 18], ion channels [19], and proteins with very different functions and cellular roles etc [20, 11].

The current vision of allostery is based on the understanding that intrinsic protein dynamics is modulated by the ligand binding which affects the structure’s function-related degrees of freedom. Hence, the coupling between the ligand binding and protein dynamics was shown to be instrumental in detecting the allosteric and catalytic sites. The strength of this link can be expressed via binding leverage [21] that measures the change in the dynamics of the binding site depending on its original plasticity, structure of the ligand, and the set of interactions that ligand makes with the binding site [22]. It was also shown that allosteric modulation takes place via communication between the allosteric and functional sites. Leverage Coupling [15, 23] serves as the measure of allosteric communication, reflecting a coherence between degrees of freedom that determine dynamics in corresponding pairs of allosteric and catalytic sites. However, binding leverage and leverage coupling do not provide a quantifiable description of the effect of binding, causality of communication, agonistic/antagonistic nature of the ligands, energetics of the regulation etc.

In this work we present a new method for quantifying the configurational work exerted in different parts of the protein as a result of the ligand binding to the allosteric site—the allosteric free energy. Causal effects of the binding is modeled in a statistical mechanical framework by estimating the per residue free energy difference between the ligand bound and ligand free proteins. An allosteric potential based on normal modes is introduced on a “per residue approximation” for evaluating the energetics of structural changes induced by the overall protein dynamics of the protein in the local environment of a residue. Specifically, starting from the protein’s set of low frequency normal modes, the allosteric free energy of the system is obtained by estimating and comparing the partition functions that describe the conformational ensembles of the ligand free and ligand bound states at the single residue level. Thus, this model allows one to analyze a change in the allosteric free energy of a residue caused by the binding of a single ligand or by the sequential binding of several effector molecules.

We used here a set of proteins collected on the basis of thorough experimental data with well documented presence of the allosteric mechanisms in the regulation of their functions. We found several ways of regulation, dependent on the structures, oligomerization states, and functions of corresponding proteins. The increase of configurational work at the functional site is an archetypal mode of allosteric communication for hetero-oligomers, *e.g.* Aspartate carbamoyltransferase ATCase. A different mode of regulation was found, for example, in NAD-dependent Malic Enzyme (NADME) where binding at the allosteric site induces overall stabilization of the structure, including the functional sites. The inhibition of activity in this protein is supported by the increased dynamics in the catalytic site caused by the binding of ATP effector. The model also successfully discriminates between the negative and positive cooperativity in allosteric regulation, where Catabolite Activator Protein (CAP) and Dihydroxyacetone Kinase (DAK) are examples of the former and Phosphofructokinase (PFK) is an example of the latter. Finally, redistribution of the configurational work in D-3-phosphoglycerate dehydrogenase (PGDH) is strongly influenced by the ring-like shape of this protein, which is an example of the role of topology in allosteric communication.

## Results

### A structure-based model of allostery

A structure-based model of protein allostery should in general address two related issues: (i) providing a quantitative description of the causal effect of allosteric ligand binding on the protein’s structural dynamics and (ii) evaluating the communication between functional and regulatory sites and the modulation of the protein’s functional activity. Thermodynamical description of the protein states, the effects of the ligand binding, and allosteric cooperativity are globally characterized by the free energy changes of the protein. If, for example, thermodynamics of the two state model of allostery is considered, the ligand free protein populates the inactive *R*_*i*_ and the active *R*_*a*_ states with the Gibbs free energy gain/loss Δ*G*(*R*_*i*_ → *R*_*a*_) = −*RT*ln[*R*_*a*_]/[*R*_*i*_], where [*R*_*a*_]/[*R*_*i*_] is the equilibrium constant for the transition *R*_*i*_ → *R*_*a*_, *R* is the ideal gas constant, and *T* is the temperature. In the event of binding of an allosteric ligand the equilibrium between active and inactive states is shifted, that is the bound states *AR*_*i*_ and *AR*_*a*_ yield the following free energy difference Δ*G*(*AR*_*i*_ → *AR*_*a*_) = −*RT*ln[*AR*_*a*_]/[*AR*_*i*_]. The global stabilizing/destabilizing effect of the active state caused by an allosteric effector is thus given by the free energy difference ΔΔ*G*(*R*_*a*_ → *AR*_*a*_) = −*RT*ln*α*, where *α* = [*AR*_*a*_][*R*_*i*_]/[*AR*_*i*_][*R*_*a*_] reflects the population shift in the active state.

Protein conformational states are the key determinants in the phenomenological thermodynamic model of allostery [14]. On the other hand, as the phenomenology of the allosteric communication involves coupling between allosteric and functional sites, structure-based models are usually characterized by the “per site/residue” descriptors [24]. The goal is to evaluate the free energy gain/loss $\Delta g_{F}\left(P\to AP\right)$ upon transition from the ligand free protein conformational ensemble (for simplicity indicated with P) to the protein conformational ensemble with the ligand *A* bound—AP. The simplest case scenario of a protein P with one allosteric site *A* and one functional site *F*, is a structure-based model that should link the ligand binding at the site *A* to the energetics of the site *F*. It is important to emphasize that this free energy is precisely the difference between the work exerted in the catalytic site of the ligand free and ligand bound protein states. We express the free energy gain/loss $\Delta g_{F}\left(P\to AP\right)$ per site as the average of independent contributions of residues *i* belonging to a site *F* such as

$$\Delta g_{F}\left(P\to AP\right)=\frac{1}{n_{F}}\sum_{i\in F}\Delta g_{i}\left(P\to AP\right)$$ (1)

with $\Delta g_{i}\left(P\to AP\right)$ the free energy gain/loss per residue caused by allosteric binding, and *n*_*F*_ is the number of residues in the site *F*. In order to estimate a relative strength of the allosteric effects, the average effect caused by the allosteric binding on all protein residues should be also evaluated:

$$\Delta g_{P}\left(P\to AP\right)=\frac{1}{n_{P}}\sum_{i\in P}\Delta g_{i}\left(P\to AP\right)$$ (2)

with $n_{P}$ the total number of residues in a protein P. Thus, the free energies $\Delta g_{i}\left(P\to AP\right)$, $\Delta g_{F}\left(P\to AP\right)$, and $\Delta g_{P}\left(P\to AP\right)$ evaluate the causality and the energetics of allosteric communication at the residue, site, and protein levels respectively. In the case of a protein with multiple allosteric sites, one can also evaluate the free energy difference

$$\Delta \Delta g_{i}\left(A^{\left(n-1\right)}P\to A^{\left(n\right)}P\right)=\Delta g_{i}\left(P\to A^{\left(n\right)}P\right)-\Delta g_{i}\left(P\to A^{\left(n-1\right)}P\right)$$ (3)

which gives the modulating effect upon sequential binding of allosteric effectors, where the $A^{\left(n\right)}P$ indicates a system with *n* ligands bound to the protein P. Thus, similarly to eqs (1) and (2) one has

$$\begin{array}{ccc} \Delta \Delta g_{F}\left(A^{\left(n-1\right)}P\to A^{\left(n\right)}P\right) & = & \frac{1}{n_{F}}\sum_{i\in F}\Delta \Delta g_{i}\left(A^{\left(n-1\right)}P\to A^{\left(n\right)}P\right)\\ \Delta \Delta g_{P}\left(A^{\left(n-1\right)}P\to A^{\left(n\right)}P\right) & = & \frac{1}{n_{P}}\sum_{i\in P}\Delta \Delta g_{i}\left(A^{\left(n-1\right)}P\to A^{\left(n\right)}P\right) \end{array}$$ (4)

**Fig 1:**
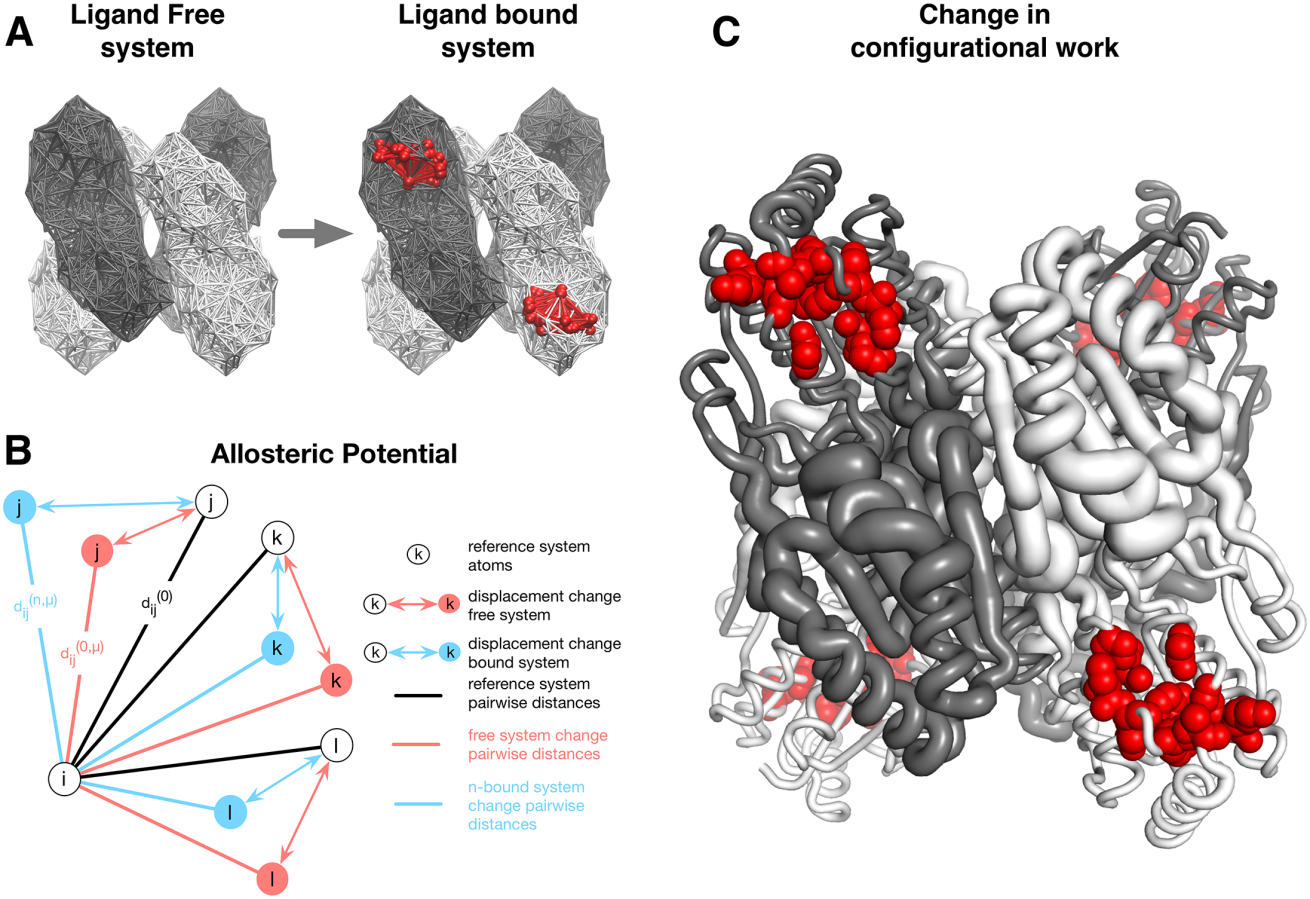
Pictorial representations of three major components of the model developed in this work. (A) The illustration of the ligand free and ligand bound states on the basis of the *C*_*α*_ harmonic approximation. The presence of a ligand at the allosteric binding site is modeled by local restraining of the residue pairs that belong to the binding site. Example of the ligated site is shown in red color. (B) Schematic representation of the concept of allosteric potential: neighbors of a residue *i* (residues *j*, *k*, *l*), assume different displacements as a consequence of the difference in the structure dynamics of the free and ligated proteins. (C) Cartoon representation of the configurational work gain/loss per residue caused by restraining of the allosteric binding sites. The radius *ρ*_*i*_ per residue*i* in the tube-like protein representation is determined by the value of the configurational work per corresponding residue, scaled according to the function *ρ*_*i*_ = (Δ*g*_*i*_ − min_*i*_ Δ*g*_*i*_)/(max_*i*_ Δ*g*_*i*_ − min_*i*_ Δ*g*_*i*_). Thus, protein portions (residues, sites, domains) represented with a thick tube show increased dynamics upon restraining of the allosteric binding site and vice versa.

Considering all the above, the main task of a structure-based model is the evaluation of the free energy gain/loss for any arbitrary residue *i* upon binding an effector molecule to an allosteric site. The method we propose for estimating the allosteric free energy $\Delta g_{i}\left(P\to AP\right)$ consists of three major components. The first component is aimed at characterizing the ligand free P and ligand bound AP protein systems by using a *C*_*α*_ harmonic model constructed on the basis of the available structures. The ligand bound system AP is obtained from the free system P by harmonic restraining of all the pairs of residues belonging to the allosteric binding site *A*. In other words, the stabilization of the allosteric binding site *A* indirectly models the presence of the actual bound molecule, affecting local dynamics of the protein. Fig 1A illustrates both ligand free and ligand bound proteins where residues and pairs of interactions belonging to the allosteric binding sites are red colored. Thus, in our definition of the ligand bound system a presence of the ligand is overwhelming, modeling all possible contacts within the binding sites. At the same time, no details of the contact interactions between ligand and binding site residues are accounted for (see Materials and Methods for the details of the harmonic model and the energy functions involved).

For exploring protein dynamics two sets of normal modes $e_{\mu }^{\left(P\right)}$, $e_{\mu }^{\left(AP\right)}$ are obtained from the harmonic protein model for the ligand free and ligand bound systems, respectively. These sets of normal modes are used as inputs for the *allosteric potential*, which is the second component of our method. The potential is used for evaluating the energetics that reflects structural differences induced by the different dynamics in the ligand free and ligand bound systems at the single residue level. The per residue allosteric potential used here is:

$$U_{i}\left(\sigma \right)=\frac{1}{2}\sum_{\mu }\varepsilon_{\mu ,i}\sigma_{\mu }^{2}$$ (5)

with the summation running over the frequency modes. The parameters *ε*_*μ*, *i*_ are characteristics of the ligand free and ligand bound systems ($\varepsilon_{\mu ,i}^{\left(P\right)}$ and $\varepsilon_{\mu ,i}^{\left(AP\right)}$, respectively), and the coefficients *σ*_*μ*_ are gaussian variables with variance 1/*ε*_*μ*, *i*_. Fig 1B provides a pictorial representation of the concept behind the per residue allosteric potential: in a ligand free and a ligand bound systems the neighbors of a residue *i* assume different displacements. The changes in dynamics of free and bound proteins is reflected in the normal modes $e_{\mu }^{\left(P\right)}$, $e_{\mu }^{\left(AP\right)}$ and, thus, in the mean elastic work that is exerted on residue *i* (see Materials and Methods for the details).

The last component of the structure-based model is a statistical mechanical approach for the calculation of the per residue allosteric free energy $\Delta g_{i}\left(P\to AP\right)$ by comparing the configurational ensembles of the ligand free and ligand bound systems. As the allosteric potential is defined at the single residue level, the ensemble of configurations of a single residue is characterized by all possible displacements assumed by its neighbors. The displacements are obtained from the linear combinations of the low frequency normal modes $e_{\mu }^{\left(P\right)}$, $e_{\mu }^{\left(AP\right)}$ for the ligand free and ligand bound systems, respectively. Thus, the calculation of the canonical partition function per residue *i* gives the allosteric free energy gain/loss black

$$\Delta g_{i}\left(P\to AP\right)=\frac{1}{2}k_{B}T\sum_{\mu }\ln\frac{\varepsilon_{\mu ,i}^{\left(AP\right)}}{\varepsilon_{\mu ,i}^{\left(P\right)}}$$ (6)

with the summation running over the low frequency modes, *k*_*B*_ is the Boltzmann constant, *T* is the temperature. Noteworthy, the free energy difference given by eq (6) is a purely configurational entropic contribution to the allosteric free energy, because the reference structure used in a harmonic model of the protein is the same in the ligand free and ligand bound systems. Therefore, the values of $\Delta g_{i}\left(P\to AP\right)$ calculated with eq (6) should be interpreted as *the difference in the amount of* *configurational work* exerted on residue *i* by its environment due to the changes in the protein dynamics caused by the binding of an allosteric ligand. The resulting free energy profiles $\Delta g_{i}\left(P\to AP\right)$ illustrate regions of the protein whose dynamics is affected by the ligand binding. Specifically, negative values of $\Delta g_{i}\left(P\to AP\right)$ reflect stabilization in the corresponding regions, positive values, on the contrary, reflect destabilization. Fig 1C shows a tube-like representation of the protein backbone with thickness rescaled as the configurational work per residue. Thus, eqs (1) and (2) constitute a general basis for the analysis of the causality and the energetics of the allosteric communication. Similarly, sequential binding of ligands can result in positive and negative cooperativity. In order to quantify that, we operate with the notion of ΔΔ*g*_*i*_ (eqs (3) and (4)), where the sign of the latter determines type of the cooperativity. It is worth noting that throughout the text—particularly in the Results section where protein cases are discussed—the terms allosteric free energy and exerted configurational work are used interchangeably.

### Different scenarios and specifics of energetics in allosteric regulation

Analyzing set of proteins below, we show how our model describes different modes of allosteric regulation. In brief, the generic procedure for the analysis of allosteric communication consists of three major steps. First, using the harmonic protein model the set of normal modes that characterize the dynamics of the ligand free and ligand bound states are obtained. The allosteric ligand binding is modeled by restraining the residues that belong to allosteric sites described in literature. Second, the allosteric potential is used to quantify the deforming effect of normal modes on the environment of protein residues. It evaluates the amount of elastic work that is exerted on protein residues of both the ligand free and ligand bound forms as a result of the proteins dynamics represented by a generic linear combination of normal modes. Third, from the allosteric potential a statistical mechanical approach is finally used for estimating the per residue partition functions. It allows one to obtain the per residue allosteric free energy gain/loss caused by effector binding. Left panels of main text figures including the proteins considered in the work and S3 Fig show profiles of the per residue allosteric free energy averaged over all homologous monomers (red curves) for the list of proteins analyzed in this work. The grey error bands around the profiles reflect the range of the work exerted per residue in each monomer (standard error), due to the structural differences between homologous monomers in the oligomeric structure. Protein surfaces (backbone in case of PGDH protein) are colored according to the detected allosteric free energy differences per residue. Protein structures are also represented with their binding sites to guide the eye in the analysis of the allosteric free energy profiles.

#### Aspartate carbamoyltransferase (ATCase)

**Fig 2:**
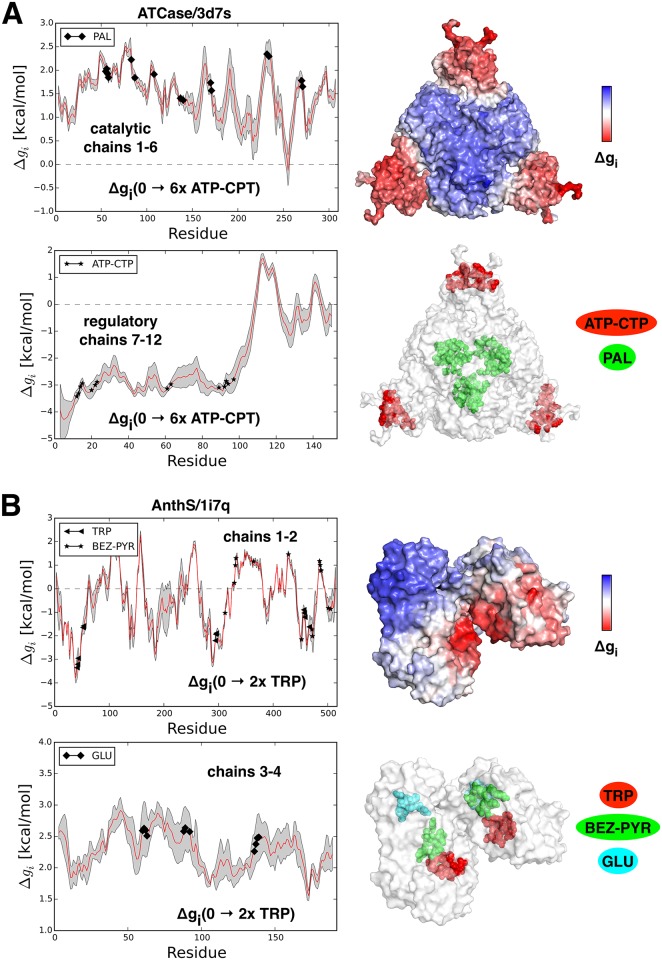
Two examples of the hetero-oligomeric allosteric proteins, the 12-mer Aspartate carbamoyltransferase ATCase (A) and 4-mer Anthranilate Synthase AnthS (B). In (A) the averaged free energy Δ*g*_*i*_(0 → 6*xATP*/*CPT*) profiles per monomer (red curves) are shown for the catalytic (PAL site) and regulatory (ATP-CTP site) chains with gray error bands. The positions of residues that belong to the allosteric and catalytic sites are marked with corresponding symbols. In the upper right panel the complex surface is colored according to the values of the conformational work per residue. The regulatory chains are strongly stabilized (red part of the Δ*g* scale), whereas the chains that carry catalytic sites yield a significant increase of configurational work (blue part of the Δ*g* scale) as a result of allosteric communication between sites. Catalytic (PAL) and regulatory (ATP-CTP) sites are shown in green and red, respectively. In (B) the averaged free energy Δ*g*_*i*_(0 → 2*xTRP*) profiles are shown for chains 1–2 (BEZ and TRP site) and chains 3–4 (GLU site) of Anthranilate Synthase AnthS. Similarly to the ATCase allosteric sites, the restraining in chains 1–2 induces high configurational work in the chains 3–4 that contain catalytic sites. Here and in the following figures containing data on proteins the red curve in the chart shows allosteric free energy profiles, the grey error band reflects the range in the amount of work exerted per residue in each monomer, because of the structural differences between homologous monomers in the oligomeric structure. Surface representations are colored according to the conformational work exerted per residues in corresponding part of the protein (red—negative values of the conformational work, showing local stabilization; blue—positive values of the work, pointing to increase of the local dynamics). We also show representative structures with color-marked locations of the corresponding ligands.

Aspartate carbamoyltransferase (ATCase) [25] is one of the best understood allosteric proteins, the hetero-dodecameric enzyme. The structure is organized in two trimers of the catalytic units and three dimers of the regulatory units (2x3+3x2). ATCase plays a crucial role in the early steps of pyrimidines biosynthesis. ATP is an allosteric activator of ATCase, which increases the reaction rate of pyrimidine synthesis. CTP is known to be an allosteric inhibitor, and high concentrations of the reaction’s end products also negatively regulate pyrimidine yield. Both activator and inhibitor share the same binding site (ATP-CTP), which is located at the outer periphery of the three regulatory dimers. The catalytic binding sites are buried in the two centrally located trimers (PAL, green, Fig 2A). PAL binding induces a large conformational change from the inactive compact state to the active expanded state. It has been shown that the transition between inactive and active states can be described by the low frequency modes responsible for the large-amplitude motion of the quaternary structure [26]. We analyzed a free energy difference for the ligand free and bound states using four available structures: two inactive forms (apo PDB ID: 3d7s and CTP bound PDB ID: 1rac) and two active forms (PAL and ATP bound form PDB ID: 7ati and the PAL bound structure PDB ID: 1d09). Upon binding to the ATP/CTP site in both inactive structures, stabilization of the allosteric site (3d7s: Δ*g*_*ATP*_(0 → 6*×ATP*) = -3.35 kcal/mol and 1rac: Δ*g*_*ATP*_(0 → 6*×ATP*) = -3.27 kcal/mol) induces an overall freezing of the regulatory monomers *R* − *mer* (3d7s: Δ*g*_*R* − *mer*_(0 → 6*×ATP*) = -2.02 kcal/mol and 1rac: Δ*g*_*R* − *mer*_(0 → 6*×ATP*) = -1.61 kcal/mol, red colored surfaces of the outer dimers, Fig 2A). This stabilization is compensated by a significant increase in the configurational work (destabilization) at the PAL binding site (3d7s: Δ*g*_*PAL*_(0 → 6*×ATP*) = 1.98 kcal/mol and 1rac: Δ*g*_*PAL*_(0 → 6*×ATP*) = 1.94 kcal/mol, blue surfaces of the two internal trimers, Fig 2A). Noteworthy, the configurational work exerted at the catalytic sites is higher than overall destabilization of the catalytic monomers *C* − *mer* (3d7s: Δ*g*_*C* − *mer*_(0 → 6*×ATP*) = 1.47 kcal/mol). At the same time, preliminary binding of the inhibitor (CTP in 1rac) practically eliminates a difference between the work exerted at the catalytic sites and the average work exerted in the whole catalytic unit (1rac: Δ*g*_*C* − *mer*_(0 → 6*×ATP*) = 1.83 kcal/mol, see also S1 Table). Despite that ligand binding to the allosteric sites in activated structures (7ati and 1d09) destabilizes the PAL binding sites (7ati: Δ*g*_*PAL*_(0 → 6*×ATP*) = 1.88 kcal/mol and 1d09: Δ*g*_*PAL*_(0 → 6*×ATP*) = 1.28 kcal/mol, see S1 Table), the whole catalytic units are being destabilized almost to the same level (7ati: Δ*g*_*C* − *mer*_(0 → 6*×ATP*) = 1.73 kcal/mol and 1d09: Δ*g*_*C* − *mer*_(0 → 6*×ATP*) = 1.18 kcal/mol). These observations show that corresponding structures (7ati and 1d09) are already in the activated state, while binding to the ATP/CTP site in the inactive state (apo form 3d7s) induces a transition from the inactive to active states.

**Table 1:** Results on allosteric causality and energetics obtained for proteins analyzed in this work. Data for only one representative structure is given in this table (for the complete list of proteins see S1 Table). First column provides protein names, degree of oligomerization and total number of residues. Second column: PDB ID of the protein and names of ligands (if not the apo form). Third column: number, name, and type (^(†)^(A) allosteric activator, (I) allosteric inhibitor, and (S) substrate) of ligated binding sites in our model. Fourth column: the mean allosteric free energy difference of the ligated binding site *A* $\Delta g_{A}\left(P\to AP\right)$ averaged over all the residues belonging to *A* in all protein monomers, and the mean allosteric free energy difference averaged over all the residues of the protein monomers (in parentheses). Fifth column: names of the binding site under allosteric regulation, typically catalytic sites. Sixth column: the mean allosteric free energy difference (or work exerted at) of the regulated binding site *F* $\Delta g_{F}\left(P\to AP\right)$ averaged over all the residues belonging to *F* in all protein monomers, and the mean allosteric free energy difference averaged over all the residues of the protein monomers (in parentheses $\Delta g_{mer}\left(P\to AP\right)$). ATCase: ^(^*^)^ and ^(^**^)^ the values corresponding to the regulatory Δ*g*_*R* − *mer*_ and catalytic Δ*g*_*C* − *mer*_ monomers, respectively. AnthS: ^(⋆)^ and ^(⋆⋆)^ the values corresponding to the monomers containing TRP Δ*g*_*TRP* − *mer*_ and GLU Δ*g*_*GLU* − *mer*_. CAP: ^(^*^)^ and ^(^**^)^ the values corresponding to the bound monomer Δ*g*_*B* − *mer*_ and free monomer Δ*g*_*F* − *mer*_.

Protein (#ch, #res)	PDB ID (ligands)	Ligated Site (A/I/S)^(*†*)^	$\Delta g_{A}(\to A)(\Delta g_{mer}(\to A))\;\text{kcal/mol}$	Regulated Site	$\Delta g_{F}(\to A)(\Delta g_{mer}(\to A))\;\text{kcal/mol}$
ATCase (2×3+3×2, 2736)	3d7s (apo)	6 × ATP/CTP (A/I)	-3.35(-2.02)^(^*^)^	PAL	1.98(1.47)^(^**^)^
AnthS (2+2, 1426)	1i7q (BEZ, PYR, GLU)	2 × TRP (I)	-2.87(-0.42)^(^*^)^	BEZ+PYR	-0.60(-0.42)^(^*^)^
				GLU	2.13(2.29)^(^**^)^
BGDH (6, 2976)	1nr7 (apo)	6 × ADP (A)	-1.51(-0.16)	NDP	0.11(-0.16)
				GLU	-0.88(-0.16)
		6 × GTP (I)	-0.32(0.61)	NDP	0.14(0.61)
				GLU	0.47(0.61)
		6 × ADP (A)	-0.64(0.60)	NDP	0.32(0.60)
		6 × GTP (I)	-0.40(0.60)	GLU	-0.01(0.60)
CAP (2, 418)	2wc2 (apo)	1 × cAMP (A)	-1.49(-0.13)^(#)^	DNA	0.88(0.24)^(##)^
				cAMP	-1.06(0.24)^(##)^
		2 × cAMP (A)	-2.37(-0.30)	DNA	0.85(-0.30)
DAHPS (4, 1401)	1ggl (PGA)	4 × PHE (I)	-2.33(0.25)	PGA	0.33(0.25)
DAK (2, 735)	3ju5 (apo)	1 × ADP (S)	-1.93(-0.72)	ADP	0.49(0.74)
		1 × ARG (S)	-0.76(-0.72)	ARG	1.03(0.74)
		2 × ADP (S)	-0.85(-0.05)	ADP	-0.85(-0.05)
		2 × ARG (S)	-0.11(-0.05)	ARG	-0.11(-0.05)
G6PD (6, 1596)	1cd5 (apo)	6 × 16G (A)	-0.04(0.31)	AGP	0.37(0.31)
NADME (4, 2232)	1efk (NAD)	4 × FUM (A)	-0.53(-0.14)	NAD	-0.14(-0.14)
		4 × ATP (S/I)	-1.42(-0.25)	NAD	0.11(-0.25)
PFK (4, 1284)	3pfk (apo)	4 × PEP/ADPa (I/A)	-0.78(0.16)	F6P	0.71(0.16)
				ADPf	-0.11(0.16)
		4 × ADPf (S/A)	-0.74(0.50)	F6P	1.74(0.50)
		4 × PEP/ADPa (I/A)	0.35(0.68)	F6P	2.22(0.68)
		4 × ADPf (S/A)	-1.08(0.68)		
PGDH (4, 1624)	1psd (SER, NAD)	8 × SER (I)	-0.58(0.03)	AKG	0.08(0.03)
				NAD	0.38(0.03)
PKA (1, 336)	1atp (ATP)	1 × ATP (I)	-4.28(-0.10)	MPD	1.97(-0.10)
PTP1B (1, 278)	2hnp (apo)	1 × 892 (I)	-3.63(-0.05)	BPM	0.36(-0.05)
SSUPRT (4, 868)	1xtu (U5P, CTP)	4 × CTP (I)	-2.89(-0.53)	U5P	0.14(-0.53)
ThrS (2, 884)	1e5x (apo)	1 × SAM (A)	-1.31(0.02)	TRS	-0.31(0.02)
				PLP	0.35(0.02)
		2 × SAM (A)	-3.67(-0.24)	TRS	-0.54(-0.24)
				PLP	0.52(-0.24)

#### Anthranilate Synthase (AnthS)

Anthranilate Synthase (AnthS) is a hetero-tetrameric enzyme catalyzing reaction that produces anthranilate (BEZ), pyruvate (PYR), and glutamate from two substrates—chorismate and L-glutamine (GLU). Substrates of this enzyme bind to two distinct sites, BEZ, PYR, and GLU (Fig 2B), mutually affecting each others activity. Tryptophan (TRP) is the allosteric down regulator for this enzyme [27] which binds at its designated site. Products BEZ and PYR share most of the residues composing their binding site, hence the BEZ-PYR is considered as a common site for both products and merged in our calculations. Two X-ray structures were analyzed for this protein: the active state structure (PDB ID: 1i7q) with three bound substrates (BEZ, PYR and GLU) and the inactivated structure (PDB ID: 1i7s) with a bound allosteric inhibitor (TRP). In both active and inactive states binding of the inhibitor leads to the stabilization of the catalytic binding site BEZ-PYR (see 1i7q in Table 1 and 1i7s in S1 Table). In the active form these effects are stronger, apparently reflecting the presence of bound substrates in the analyzed active structure (1i7q).

#### Bovine Glutamate Dehydrogenase (BGDH)

**Fig 3:**
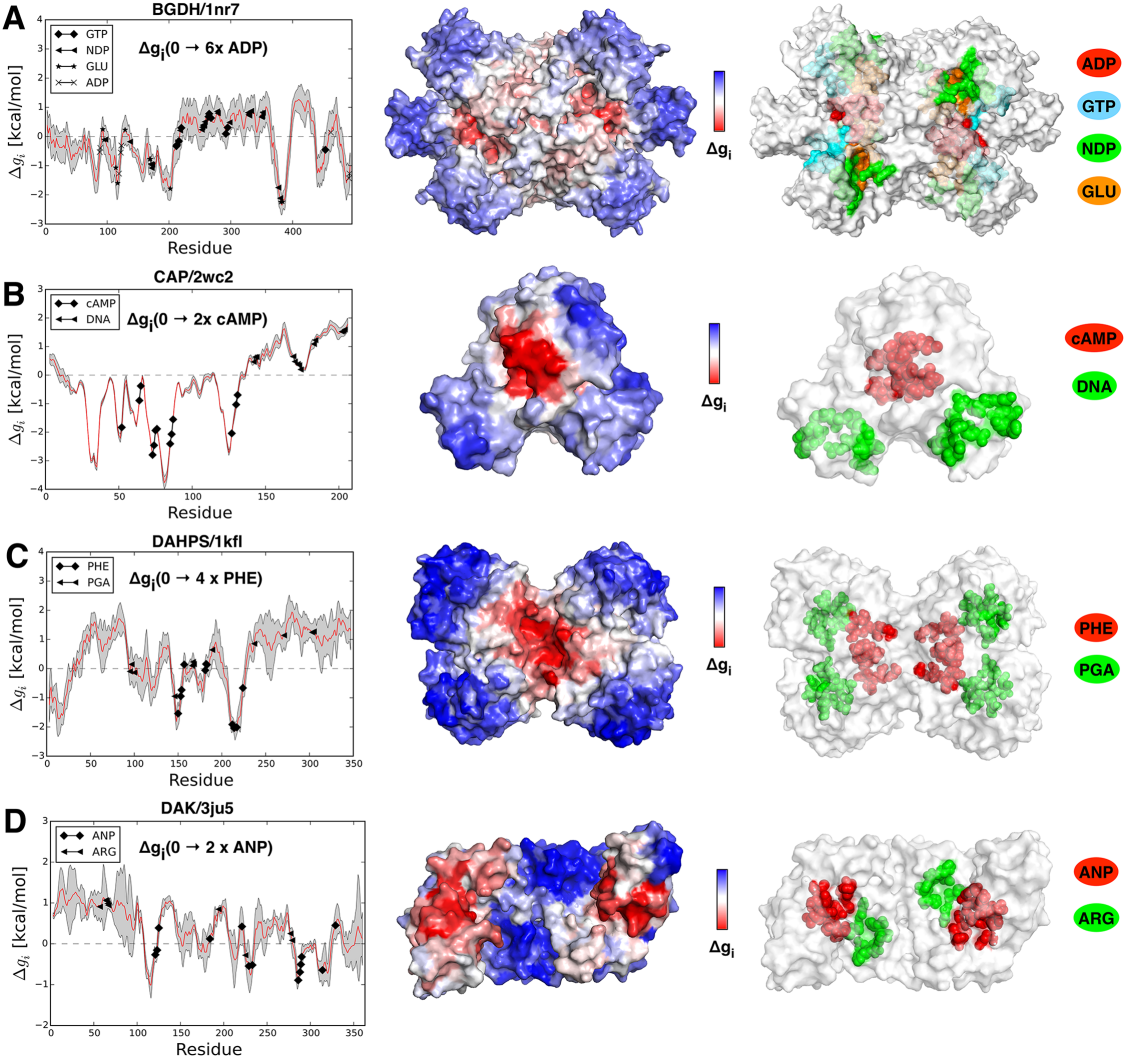
Free energy profiles and structural representatives. (A) bovine glutamate dehydrogenase BGDH free energy profile Δ*g*_*i*_(0 → 6*xADP*) with marked residue positions of the GTP, NDP, GLU, and ADP binding sites. (B) Catabolite activator protein (CAP) free energy profile Δ*g*_*i*_(0 → 2*xcAMP*) with marked locations of the cAMP and DNA binding sites. (C) The 3-Deoxy-D-arabinoheptulosonate 7-phosphate synthase DAHPS free energy profile Δ*g*_*i*_(0 → 4*xPHE*) with marked positions of the PHE and PGA binding sites. (D) The dihydroxyacetone kinase DAK free energy profile Δ*g*_*i*_(0 → 2*xANP*), ANP and ARG are ligands.

With about 3000 residues the bovine glutamate dehydrogenase (BGDH) is the largest complex analyzed in our protein list (see Fig 3).

**Fig 4:**
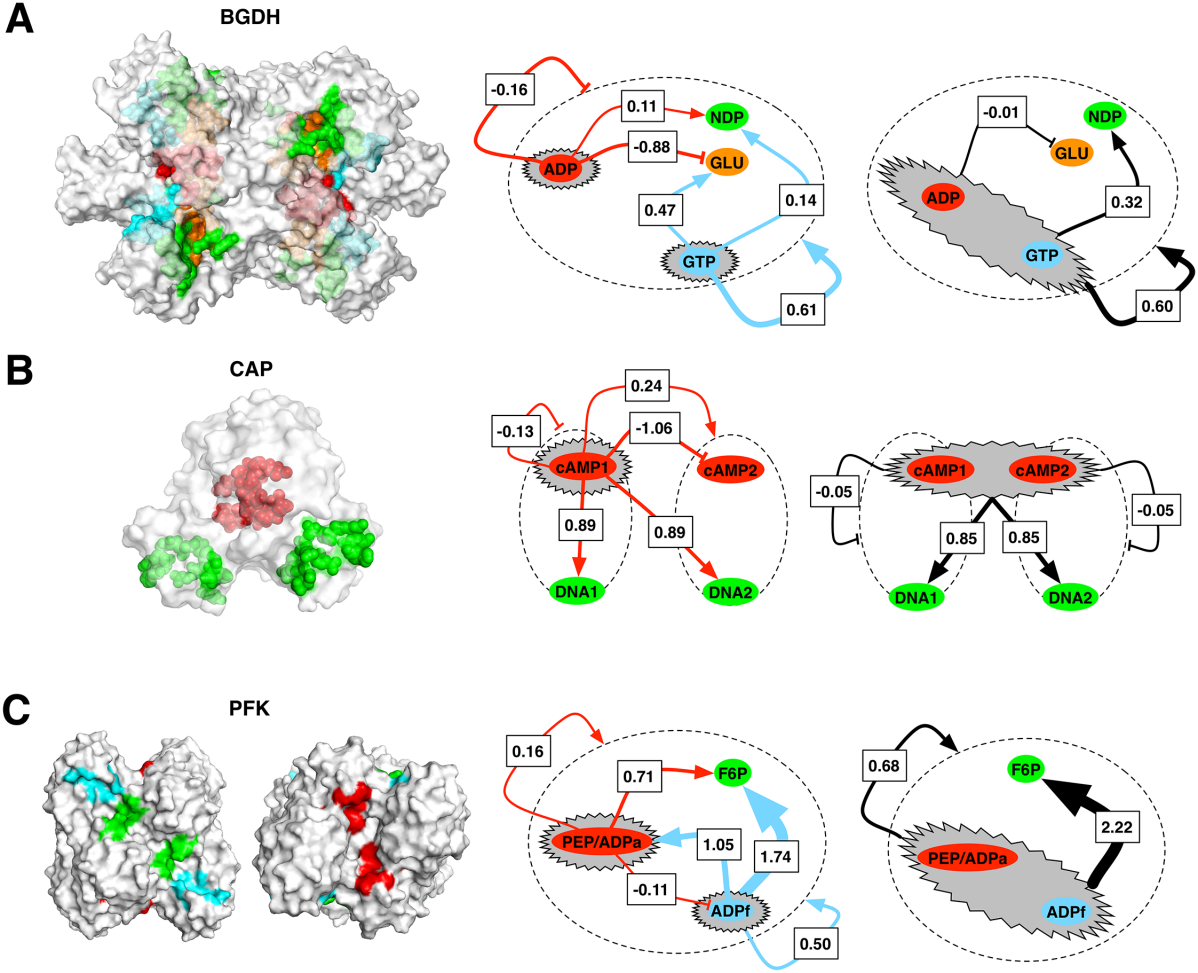
Schematic representation of the details of allosteric communication. (A) bovine glutamate dehydrogenase, BGDH; (B) catabolite activator protein, CAP; (C) phosphofructokinase, PFK. Structures show locations of the binding sites, which have node shapes in the diagrams on the right. Grey ovals show ligated allosteric sites in corresponding modes of regulation. Arrows illustrate the causal relations between the allosteric and functional sites. Numbers on the arrows provide the allosteric free energy (or work exerted, in kcal/mol) at the catalytic site *F* as a result of binding at the allosteric site, $\Delta g_{F}\left(P\to AP\right)$. (A) BGDH. Simultaneous binding of *ADP* and *GTP* is compared to independent ones. In both PFK and BGDH schemes the dashed line ovals represent the whole protein complex. (B) CAP. There are two possible scenarios of regulation, depending on whether one (1*xcAMP*) or two (2*xcAMP*) allosteric ligands are bound. The dashed line ovals represents the protein monomers. (C) PFK. The mode of regulation in which both*PEP*/*ADPa* and *ADPf* ligands are independently bound is compared to the case in which both effector and substrate are simultaneously bound, revealing the modulating role of *ADPf* in the configurational work exerted at the *F*6*P* site.

This homo-hexamer protein catalyses the oxidative deamination of l-glutamate to 2-oxoglutarate. It is up-regulated by ADP and down-regulated by GTP. The coenzyme NADP+ that binds in the NAD site is an antagonist. Catalytic sites (where substrates GLU and NDP bind) are located very close to each other in the catalytic cleft. ADP is found in the active and inactive conformations. It supposedly favors transition between two states by facilitating the opening of the catalytic cleft [28]. GTP binds only to the inactive conformation [29]. Calculations were performed on the three structures: the apo form (PDB ID: 1nr7) and two forms with a bound allosteric ligand, GTP (PDB ID: 1hwz) and ADP (PDB ID: 1nqt), respectively. In particular, we compared allosteric free energy in the ligand free state with those in structures with bound allosteric ligands (GTP or ADP). In the active apo form (1nr7) we observed that significant decrease (Δ*g*_*ADP*_(0 → 6*×ADP*) = −1.51 kcal/mol) of the configurational work at the activator site ADP induces a small overall stabilization of the protein (Δ*g*_1*nr*7_(0 → 6*×ADP*) = -0.16 kcal/mol)—see Fig 3A for the corresponding allosteric free energy profile. As a result, GLU site is strongly stabilized (Δ*g*_*GLU*_(0 → 6*×ADP*) = -0.88 kcal), whereas NDP site yields lightly increased configurational work (Δ*g*_*NDP*_(0 → 6*×ADP*) = 0.11 kcal). If the inhibitor (GTP) is bound, the active state is destabilized Δ*g*_1*nr*7_(0 → 6*×GTP*) = 0.61 kcal, while the NDP and GLU substrate sites are destabilized less than the units containing them (Table 1). Free energy changes associated with the substrate binding sites point to the activation of the protein via relaxation of the NDP binding site. Another active form of the protein, with ADP activator bound (1nqt), shows qualitatively similar results. High structural similarity (RMSD~0.4 Å) between the apo (1nr7) and holo (1nqt) active forms of BGDH hints that binding of the ADP activator mostly stabilizes the inherently active apo form of the protein. The results of calculations for the inactive holo form (1hwz) with bound inhibitor (GTP) and both substrates (NDP and GLU) are very different compared to those obtained for the active forms. Binding of the activator results in the overall stabilization of the whole structure (Δ*g*_1*hwz*_(0 → 6*×ADP*) = −0.41 kcal/mol) along with quite pronounced freezing of the catalytic sites. These observations are in agreement with the role of activator as a stabilizer of the active apo form (in 1nr7 and 1nqt structures). Fig 4A illustrates complex mechanisms of the BGDH regulation (left scheme in Fig 4A). The right scheme in Fig 4A illustrates a toy experiment in which both allosteric regulators are bound (ADP and GTP), which shows the prevailing effect of the inhibitor (see Table 1). Interestingly, the pronounced changes in the dynamics of the structure reflect the conformational changes caused by the binding of the inhibitor, whereas binding of the activator only stabilizes the apo form.

#### Catabolite Activator Protein (CAP)

Catabolite Activator Protein (CAP), dimeric transcription factor, is a very well studied example of DNA-binding activity modulation by means of what is commonly understood as dynamically driven (or governed by conformational entropy) allostery [30]. Allosteric regulation is performed via negatively cooperative cAMP-binding in two binding sites (cAMP)—one for each protein monomer. NMR studies have clearly shown that binding at only one of the two sites increases fluctuations in the structure without changing its conformation, whilst binding at both sites greatly reduces fluctuations in the whole complex [30]. We studied all three available CAP structures: the apo form (PDB ID: 2wc2), the structure with one allosteric site ligated (PDB ID: 1run), and the form with both allosteric sites ligated and DNA bound (PDB ID: 1o3q). The apo form was obtained as a mean conformer from the 20-structure bundle NMR data [31]. We applied our method in two steps by considering the allosteric free energies of states with one and with both cAMP sites ligated, and by comparing them with allosteric free energies in the apo form. Differences in configurational work are estimated for the allosteric sites and for the DNA-bound region (Table 1 and Fig 3B). First, we consider the model based on the apo structure (2wc2). If a single cAMP site is ligated, it stabilizes the whole bound monomer (Δ*g*_*B* − *mer*_(0 → 1*×cAMP*) = -0.13 kcal/mol), along with a significant destabilization (Δ*g*_*F* − *mer*_(0 → 1*×cAMP*) = 0.24 kcal/mol) of the free monomer, as well as destabilization (Δ*g*_*DNA*_(0 → 1*×cAMP*) = 0.88 kcal/mol) of the DNA-binding regions. If the cAMP sites are occupied in both monomers the overall conformational work of the whole protein is further reduced Δ*g*_2*wc*2_(0 → 2*×cAMP*) = -0.30 kcal/mol, reflecting the loss of structural flexibility in qualitative agreement with the experimental observations. The conformational work at the DNA-binding regions (Δ*g*_*DNA*_(0 → 2*×cAMP*) = 0.85 kcal/mol) is similar to the value observed for the structure with the only one allosteric site ligated. The difference ΔΔ*g*_2*wc*2_ = Δ*g*_2*wc*2_(0 → 2*×cAMP*) − Δ*g*_2*wc*2_(0 → 1*×cAMP*) = −0.54 kcal/mol is consistent with the known negative cooperativity of CAP upon cAMP multiple binding. In the other structures with cAMP bound (1run) and both cAMP and DNA bound (1o3q) a qualitatively similar picture is observed. Analysis of these two forms shows that binding of both cAMPs relaxes the DNA binding site (an increase in flexibility) and facilitates DNA binding. If DNA is already bound, cAMPs binding provides further stabilization of the DNA binding site. In Fig 4B two schematic graphs give a pictorial summary of CAP’s regulation mechanisms in the situations of single (left) and double (right) ligated cAMP binding sites.

#### 3-deoxy-D-arabinoheptulosonate 7-phosphate Synthase (DAHPS)

3-deoxy-D-arabinoheptulosonate 7-phosphate synthase (DAHPS) is a key enzyme involved in the biosynthesis of aromatic amino acids such as phenylalanine, tyrosine, and tryptophan. The amino acid products work as allosteric effectors that provide a feedback regulation and inhibit the catalytic activity. We study two DAHPS forms: inactive (PDB ID: 1kfl) and active (PDB ID: 1gg1). The former is crystallized in presence of the allosteric inhibitor-product L-phenylalanine (allosteric site PHE) and substrate phosphoenolpyruvate (active site PGA); the latter—in the presence of the substrate only. The over-stabilization of the PHE site (Δ*g*_*PHE*_(0 → 4*×PHE*) = −2.33 kcal/mol) in the active form induces an increase of configurational work at the PGA functional site (Δ*g*_*PGA*_(0 → 4*×PHE*) = 0.33 kcal/mol), which is higher than the overall increase of the mean configurational work Δ*g*_1*gg*1_(0 → 4*×PHE*) = 0.25 kcal/mol (see Fig 3C for the corresponding allosteric profiles). This suggests that the inhibition effect caused by PHE binding may facilitate the substrate release from the catalytic site. This observation is in a qualitative agreement with the results obtained for the inactive form (1kfl), where all the observed effects are less pronounced (S1 Table).

#### Dihydroxyacetone Kinase (DAK)

The dihydroxyacetone kinase (DAK) is a protein kinase that catalyzes the phosphoryl transfer between phosphagen and ADP necessary for the energy exchange in cell metabolism. The enzymatic activity in DAK is homotropically regulated by its substrate that works as a negatively cooperative inhibitor [32]. There is an interaction between active sites of two monomers, which is archetypal for homo-dimeric enzymes with negative cooperativity [32]. In particular, ligand binding in one monomer affects the active site of the other monomer. Two ligands, substrate L-arginine (site ARG) and product ADP, bind to the catalytic site of the protein. Bound to one of the monomers, both ligands can also be regarded as allosteric effectors for another part of the protein. We studied the active form (PDB ID: 3ju5) and the inactive one (PDB ID: 3ju6), which contains ARG and the ADP analog—ANP (Table 1 and Fig 3D). Occupation of sites ADP and ARG in one monomer of the active form rigidify it (Δ*g*_*B* − *mer*_(0 → 1*×*(*ADP* + *ARG*)) = −0.72 kcal/mol), increasing the configurational work in the free monomer (Δ*g*_*F* − *mer*_(0 → 1*×*(*ADP* + *ARG*)) = 0.74 kcal/mol). As a result, the free energy loss by the ligated allosteric site monomer is compensated by the work exerted at the active site monomer. When both active sites are ligated the overall configurational work of the whole protein is greatly reduced (Δ*g*_3*ju*5_(0 → 2*×*(*ADP* + *ARG*)) = −0.05 kcal/mol). The difference, ΔΔ*g*_3*ju*5_(1*×*(*ADP* + *ARG*)→2*×*(*ADP* + *ARG*)) = Δ*g*_3*ju*5_(0 → 2*×*(*ADP* + *ARG*)) − Δ*g*_3*ju*5_(0 → 1*×*(*ADP* + *ARG*)) = −0.79 kcal/mol, reflects a typical phenomenology of the negative cooperativity, and it is consistent with the inhibition mechanisms proposed elsewhere [32]. The results obtained for the inactive form (1ju6) are in qualitative agreement (S1 Table) with those obtained for the active structure.

#### Glucose-6-phosphate Dehydrogenase (G6PD)

**Fig 5:**
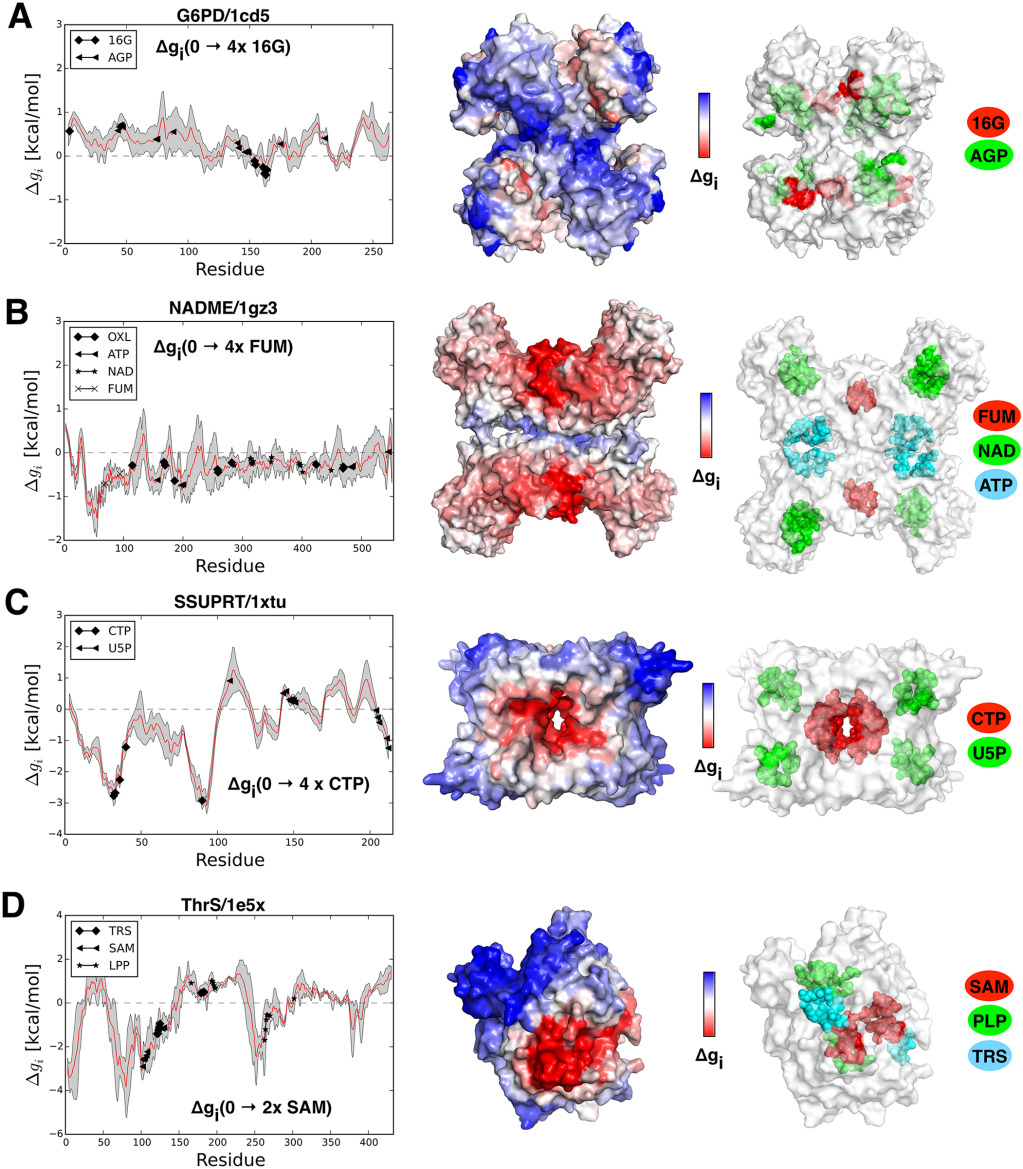
Free energy profiles and structural representatives of (A) G6PD, (B) NADME, (C) SSUPTR, and (D) ThrS. (A) 6-mer glucose-6-phosphate dehydrogenase G6PD free energy profile Δ*g*_*i*_(0 → 6*x*16*G*) with marked locations of the 16G and AGP binding sites. (B) Malate dehydrogenase (decarboxylating) enzyme NADME free energy profile Δ*g*_*i*_(0 → 4*xFUM*) with indicated positions of ATP, NAD and FUM binding sites. (C) Sulfolobus solfataricus uracil phosphoribosyltransferase SSUPTR free energy profile Δ*g*_*i*_(0 → 4*xCTP*) with marked CTP and U5P binding sites. (D) Threonine synthase ThrS free energy profile Δ*g*_*i*_(0 → 2*xSAM*) with shown locations of the TRS, SAM and LPP binding sites.

Hexaoligomeric glucose-6-phosphate dehydrogenase (G6PD) catalyzes a regulatory step of N-acetylglucosamine catabolism, producing fructose 6-phosphate and ammonia via isomerisation and deamination of the glucosamine 6-phosphate [33]. Activity of G6PD is regulated by the binding of N-acetylglucosamine-6-phosphate, which acts as an allosteric activator. We studied three available forms of the protein: the inactive apo form (PDB ID: 1cd5), the ligated form (PDB ID: 1hor) with a competitive inhibitor (site AGP) bound to the active site, and the active form (1hot) with bound allosteric ligand (site 16G). When applied to the apo form of the protein our model shows that binding at the allosteric sites (16G) does not result in their stabilization (Δ*g*_16*G*_(0 → 6*×*16*G*) = -0.04 kcal/mol). At the same time, we detected an overall destabilization of the whole complex (Δ*g*_1*cd*5_(0 → 6*×*16*G*) = 0.31 kcal/mol) and the functional sites (Δ*g*_*AGP*_(0 → 6*×*16*G*) = 0.37 kcal/mol). The destabilization effect Δ*g*_*i*_(0 → 6*×*16*G*) is illustrated in Fig 5A. The large errors per monomer are also evident, reflecting the asymmetry in the X-ray structure. The forms with competitive inhibitor (1hor) and with activator (1hot) show different behavior with respect to the apo form, but they yield qualitatively similar behavior between themselves. This similarity reflects the fact that both forms 1hot and 1hor are likely in the same conformational state, which is different from that of the apo form. It has been shown that low frequency modes explain the allosteric transition for G6PD from the apo form to the active one [21], and the destabilization of the protein is apparently a result of large quaternary structure reorganization caused by the binding of the allosteric ligand [34].

#### NAD-dependent Malic Enzyme (NADME)

Malate dehydrogenase (decarboxylating) enzyme (NADME) is a homo-tetrameric oxidoreductase that catalyzes decarboxylation of L-malate to pyruvate and the concomitant reduction of the cofactor NAD+ or NADP+. ATP works as a competitive inhibitor of the NAD active site, and it can also bind at the tetrameric interface (site ATP). Fumarate binds at the dimeric interface (site FUM), working as an allosteric activator [35]. We considered two forms of the NADME protein: the open active form (1efk) ligated with NADP+ substrate at the NAD binding site, and the closed inactive form (1gz3) in complex with the competitive inhibitor ATP and activator FUM. Binding at the FUM site in the model based on the open form (1efk) has a mild stabilizing effect on the whole complex (Δ*g*_1*efk*_(4*×FUM*) = −0.14 kcal/mol), as well as on the active site NAD (Fig 5B). Interestingly, restraining the ATP site induces an overall stabilization of the protein (Δ*g*_1*efk*_(4*×ATP*) = −0.25 kcal/mol), but a mild increase in configurational work at the NAD active site (Δ*g*_*NAD*_(4*×ATP*) = 0.11 kcal/mol). One can presume, therefore, that the allosteric activation by the fumarate is coupled with a stabilization of the active site, thus providing a proper binding of a substrate. On the other hand, the work increasing at the active site caused by the ATP binding might partially induce inhibition as it would facilitate the substrate release. Similar qualitative results were obtained in the model based on the inactive form (see 1gz3 in S1 Table). The availability of the NADME’s apo form would be important for finalizing the scenario of NAMDE’s regulation.

#### Sulfolobus Solfataricus Uracil Phosphoribosyltransferase (SSUPTR)

The sulfolobus solfataricus uracil phosphoribosyltransferase (SSUPTR) is a homo-tetramer that catalyzes the transformation of 5-phosphate-alpha-1-diphosphate and uracil into uridine 5’-monophosphate (site U5P) and diphosphate. The complex is allosterically inhibited by CTP (site CTP). It has been suggested that CTP binding and consequent structural changes of the complex stabilize the flexible loop in a closed conformation [36]. Four CTP binding sites are located at the central interface between two dimers (see Fig 5C), while four functional sites (U5P) are placed at the outer corners of the complex. Two structures were considered in our model: the form with bound substrate U5P and effector CTP (PDB ID: 1xtu), and with only substrate bound to the protein (PDB ID: 1xtt). Restraining the allosteric sites in the 1xtu form induces significant stabilization of the whole complex (Δ*g*_1*xtu*_(0 → 4*×CTP*) = -0.53 kcal/mol), as well as an increase in the configurational work at the functional sites. An increase in the configurational work at the active sites, along with unchanged overall protein stability in the other form (1xtt, see S1 Table) suggest that allosteric inhibition acts by decreasing plasticity of the active site.

#### Threonine Synthase (ThrS)

Threonine synthase (ThrS) is a homo-dimeric enzyme that is in charge of the final steps of the threonine synthesis (TRS), using the pyridoxal-L-phosphate PLP substrate. It has been shown that allosteric activation caused by the S-adenosylmethionine (SAM) binding triggers a large reorganization of the substrate site in one monomer that induces a conformation change of PLP to an active conformation [37]. Restraining SAM binding sites in a model based on the protein’s apo form (1e5x) does not affect an overall dimer stability, while it increases work at both PLP sites (Δ*g*_*PLP*_(0 → 1*×SAM*) = 0.35 kcal/mol). Restraining both SAM sites causes significant stabilization of the dimer (Δ*g*_1*e*5*x*_(0 → 2*×SAM*) = −0.24 kcal/mol), and, at the same time, it increases configurational work at the PLP sites (Δ*g*_*PLP*_(0 → 2*×SAM*) = 0.52 kcal/mol, Fig 5D). A mild positive cooperativity at the PLP site (ΔΔ*g*_*PLP*_(1*×SAM* → 2*×SAM*) = 0.17 kcal/mol) was also observed.

#### D-3-phosphoglycerate Dehydrogenase (PGDH)

**Fig 6:**
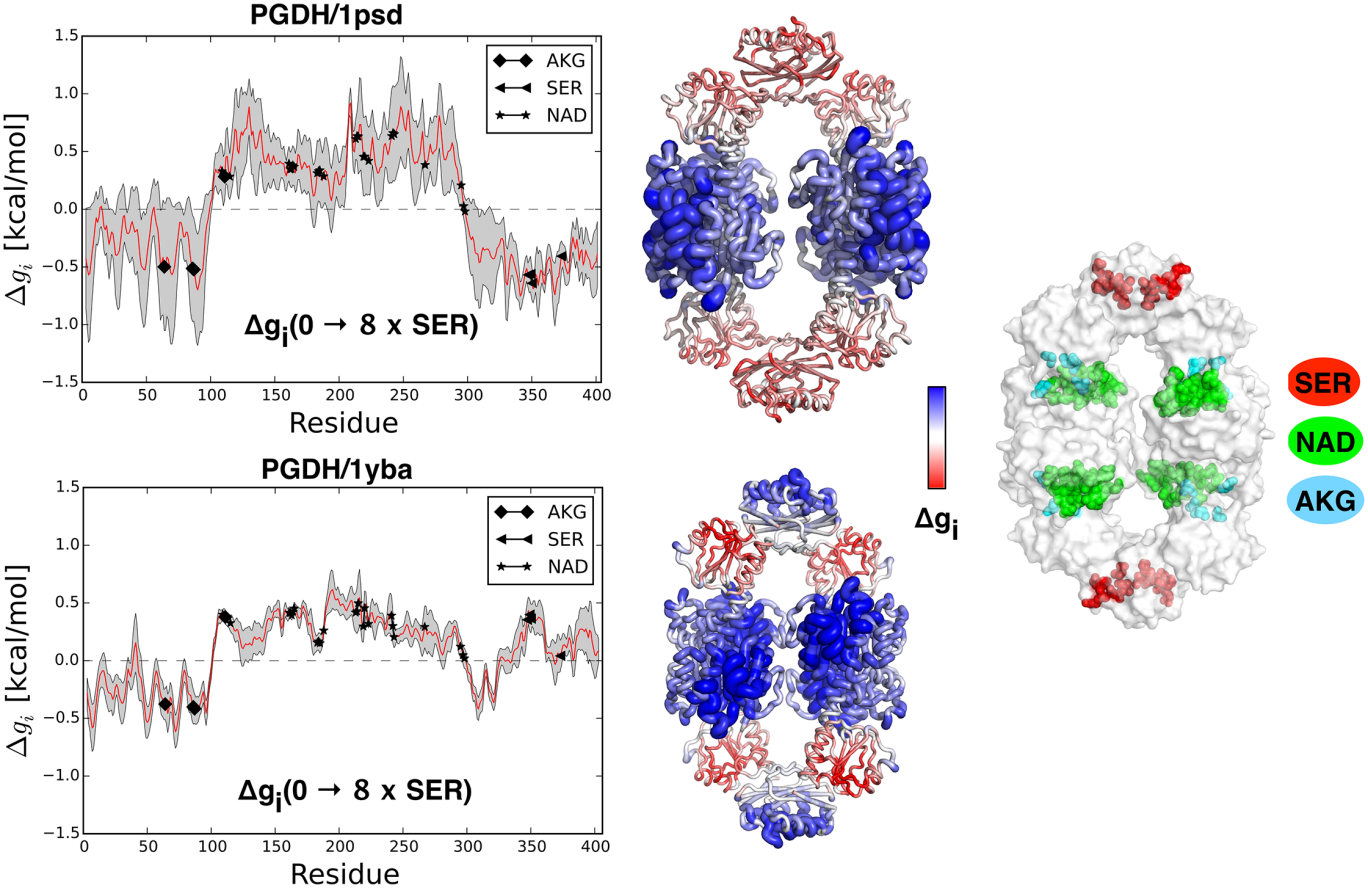
Free energy profiles and structural representations of the D-3-phosphoglycerate dehydrogenase PGDH. The profiles Δ*g*_*i*_(0 → 8*×SER*) are based on calculations performed on two protein forms, 1psd and 1yba, respectively. Protein structures are shown using a colored tube-like representation with both colors, and radius of the tube scaled according to the configurational work exerted per corresponding residue (the radius *ρ*_*i*_ per residue *i* is scaled according to the function*ρ*_*i*_ = (Δ*g*_*i*_ − min_*i*_ Δ*g*_*i*_)/(max_*i*_ Δ*g*_*i*_ − min_*i*_ Δ*g*_*i*_)). Structures in the middle represent two forms of the protein analyzed in this work: inactive (1psd, top) and active (1yba, bottom). The structure in the right panel illustrates locations of the protein’s ligands: SER (red), NAD (green), and AKG (blue).

The D-3-phosphoglycerate dehydrogenase (PGDH) is a ring-shaped tetrameric enzyme that catalyzes formation of 3-phosphohydroxypyruvate from 3-phospho-D-glycerate with NAD as a cofactor. PGDH is allosterically regulated by serine as a cooperative inhibitor. Binding of the pair of serines to dimer interfaces inhibits PGDH, affecting mainly the rate of catalytic reaction [38]. We analyzed two available X-ray forms: the first structure includes the allosteric inhibitor SER and cofactor NAD (PDB ID: 1psd); the second structure (PDB ID: 1yba) contains substrates AKG, NAD bound at the catalytic site. Upon binding to the allosteric sites in the inhibited structure, the tetramer is weakly destabilized (Δ*g*_1*psd*_(0 → 8*×SER*) = 0.03 kcal/mol), while the substrate sites, in particular NAD (Δ*g*_*NAD*_(0 → 8*×SER*) = 0.38 kcal/mol), yield some increase of the configurational work. As shown in Fig 6, increase of the work exerted at four central domains of the tetramer is compensated by the over-stabilization of the peripheral domains where the allosteric binding takes place (see S1 Table). Qualitatively similar results for overall stability of the protein and for the work exerted at the functional sites are obtained by restraining the allosteric sites in the active form (1yba). However, restraining of the allosteric sites in the active form of the protein does not result in stabilization of the domains that contain them (S1 Table). On the contrary, the work exerted in the corresponding domains of the inhibited form (1psd) is positive (bottom structure in Fig 6). Additionally, the work exerted at the functional sites of the active form is lower than the one obtained for the inactive structure. Comparison of the results obtained for inactive and active forms shows that the PGDH’s active state is supported by the global dynamics of the whole structure rather than by increase of the local dynamics in the catalytic monomers.

#### Phosphofructokinase (PFK)

**Fig 7:**
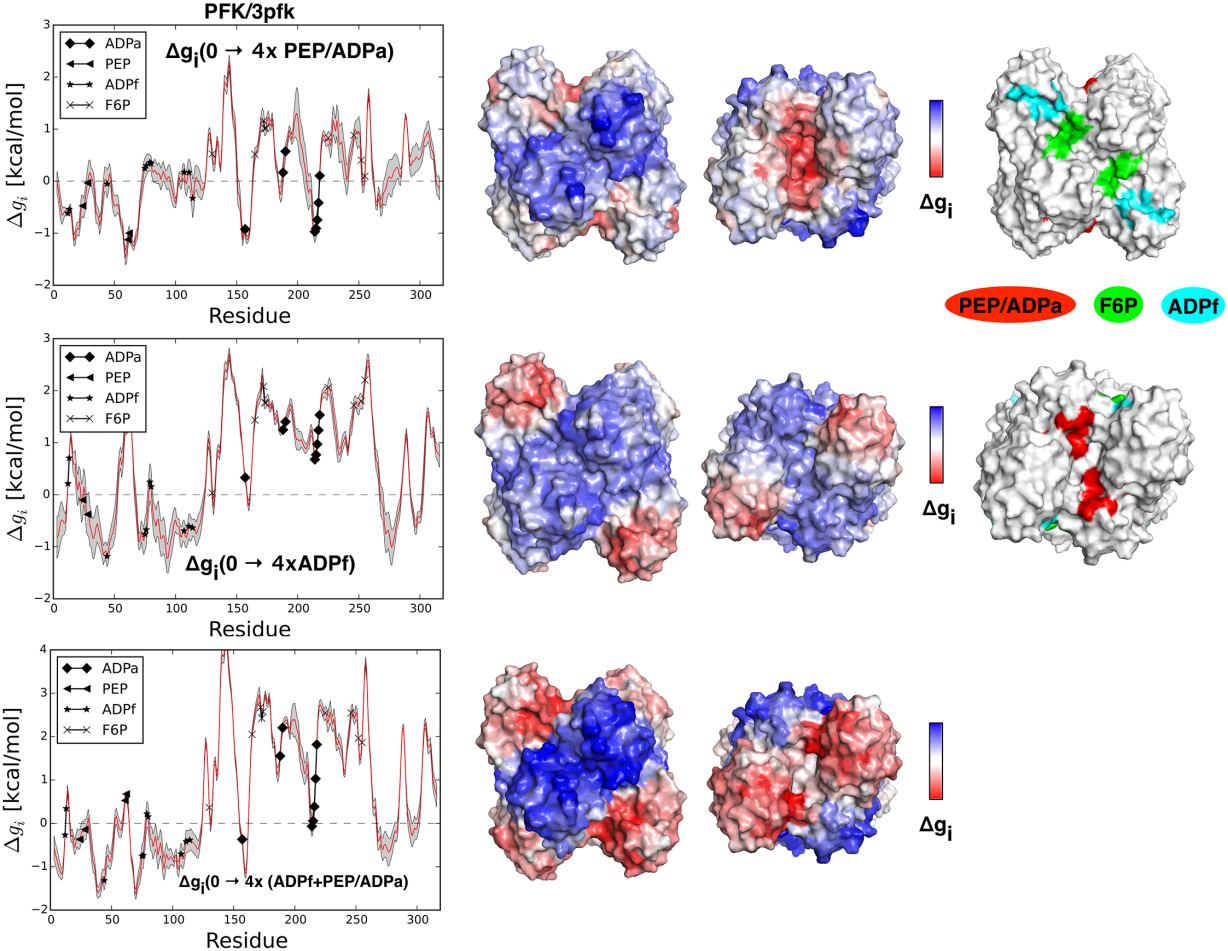
Free energy profiles and representative structures of Phosphofructokinase PFK upon restraining its various sites. All the results shown here are obtained from the analysis of the protein apo form (PDB ID: 3pfk). Three situations are shown: the inhibitor/activator PEP/ADPa is bound Δ*g*_*i*_(0 → 4*xPEP*/*ADPa*); the substrate (ADPf) is bound Δ*g*_*i*_(0 → 4*xADPf*); and both (PEP/ADPa and ADPf) ligands are bound Δ*g*_*i*_(0 → 4*x*(*PEP*/*ADPa* + *ADPf*)). The free energy profiles are shown with the colored protein surfaces according to the configurational work exerted per residue (middle column), and with representative structures where the locations of the bound ligands are marked by different colors (right column).

Phosphofructokinase (PFK) is an important regulatory enzyme of glycolysis and is one of the best studied allosteric proteins [39]. PFK’s substrate is fructose-6-phosphate (site F6P) and its four subunits are controlled by the variety of activators and inhibitors. The protein is allosterically activated by ADP and inhibited by phosphoenolpyruvate PEP whose binding sites essentially overlap (Fig 7, common site PEP/ADPa). Another ADP binding site (site ADPf) is located in the vicinity of the functional one (F6P) at the dimer-dimer interface. We analyzed three PFK structures: the apo form (PDB ID: 3pfk), the structure with bound ADPa and ADPf (PDB ID: 4pfk), and the structure with bound inhibitor PEP (PDB ID: 6pfk). Both the apo and the ADP-bound forms are the active ones, and their high structural similarity indicates that binding of the activator stabilizes the apo conformation. Transition from the active to the inactive form is characterized by the rotation of tetramer subunits [40], which can be fairly well described by the low frequency normal modes [21]. Upon binding at the PEP/ADPa site the whole protein is only weakly destabilized (Δ*g*_3*pfk*_(0 → 4*×PEP*/*ADPa*) = 0.16 kcal/mol), while the F6P sites yield an increase of the mean configurational work (Δ*g*_*F*6*P*_(0 → 4*×PEP*/*ADPa*) = 0.71 kcal/mol) along with mild stabilization of the functional ADPf binding sites (Δ*g*_*ADPf*_(0 → 4*×PEP*/*ADPa*) = −0.11 kcal/mol). Interestingly, having the only substrate ADPf binding sites ligated not only causes an overall destabilization of the protein (Δ*g*_3*pfk*_(0 → 4*×ADPf*) = 0.50 kcal/mol), but it also results in a significant increase of configurational work at the F6P site (Δ*g*_*F*6*P*_(0 → 4*×ADPf*) = 1.74 kcal/mol). Binding at both PEP/ADPa and ADPf sites also leads to a major increase of configurational work at the F6P site (Δ*g*_*F*6*P*_(0 → 4*×*(*PEP*/*ADPa* + *ADPf*)) = 2.22 kcal/mol). Fig 7 contains the allosteric free energy profiles in cases of binding at the PEP/ADPa, ADPf, and PEP/ADPa+ADPf sites, respectively. The effect of occupying PEP/ADPa and functional ADPf sites unravels the double-fold role of the PEP/ADPa site. First, it can provide an inhibition upon binding the antagonist via decrease of the configurational work at the functional ADPf site (Δ*g*_*ADPf*_(0 → 4*×PEP*/*ADPa*) = −0.11 kcal/mol). At the same time, it can work as a modulator increasing configurational work in the functional sites upon binding the ADP to both PEP/ADPa and functional ADPf sites (ΔΔ*g*_*F*6*P*_(4*×PEP*/*ADPa* → 4*×*(*PEP*/*ADPa* + *ADPf*)) = 1.51 kcal/mol). Modulating activity of the PEP/ADPa site agrees with an early detected positive cooperativity in binding of two substrates (F6P and ADP) in presence of the PEP [41]. PFK’s regulation is schematically illustrated in Fig 4C. Both cases, with the binding sites PEP/ADPa (inhibition) and ADPf (activation) individually ligated, and with effectors bound to both sites (positive cooperativity), are shown in figure. The other forms of the proteins show qualitatively similar results (S1 Table).

#### Protein Kinase A (PKA) and protein tyrozine phosphatase 1B (PTP1B)

These are two small monomeric proteins for which we analyzed the apo forms and structures with bound inhibitors (see the allosteric free energy profiles in S3 Fig). In both cases we found that binding of the inhibitor causes relaxation in the catalytic site. One can hypothesize, therefore, that inhibition in these proteins happens via the release of the substrate from the destabilized catalytic site.

### Brief overview of the analyzed proteins

There are different scenarios of allosteric regulation in 14 proteins analyzed in this work. Both activation and inhibition modes are observed for BGDH and PFK; only activation modes for ATCase, CAP, DAK, G6PD, NADME, and ThrS; and only inhibition modes for AnthS, DAHPS, SSUPTR, PGDH, PKA, and PTP1B. In CAP and DAK activation modes are coupled with negative cooperativity; in ThrS—with positive cooperativity; and in PFK there are both modes of regulation and positive cooperativity. Since there is no experimental data on free energy changes associated with allosteric signaling at a single residue level, the allosteric free energy obtained in our model can be only qualitatively compared with experimental observations. Computationally obtained free energy changes allow one to conclude on the modulation of dynamics in the catalytic sites under regulation. Positive and negative values of Δ*g* reflect increased/decreased dynamics in the corresponding catalytic sites. We found that changes in the site dynamics are always opposite in proteins with both modes of regulation (BGDH and PFK). Our data also indicated positive cooperativity in PFK. In all cases of activation except BGDH, the activation mode is coupled with increased dynamics at the catalytic site. A combination of the activation with negative cooperativity in CAP and DAK and with positive cooperativity in ThrS was detected. An increase in catalytic site dynamics in BGDH, DAHPS, SSUPRT, PKA, and PTP1B provides an inhibition mode. In AnthS and PFK inhibition is based on stabilization of the catalytic sites (decreased dynamics). We found that either increase or decrease in dynamics of the catalytic site can be associated with activation or inhibition. For example, increase of dynamics can result in activation favoring the binding of substrate, or contribute to inhibition supporting release of the substrate. At the same time, a decrease in dynamics can stabilize the active state of the catalytic site, or can prevent binding of the substrate (inhibition). As a result, any change in a catalytic site’s dynamics is indicative of the regulation, albeit the mode of regulation should be further explored. Another important aspect that should be studied in the future is the issue of agonists/antagonists that bind to the same allosteric site. For example, opposite modes of the regulation were detected, depending on the bound allosteric ligand in ATCase and PFK. Current and future work in this direction includes development of the atomic resolution model that can determine agonistic/antagonistic nature of individual residues and their combinations within the same binding site. Merged with state-of-the-art experimental techniques, e.g. disulfide trapping [42], this approach could provide a foundation for design of allosteric effectors with required agonistic/antagonistic activity [22].

## Discussion

The rise in interest in allosteric processes and, as a consequence, turning the focus to the design of allosteric drugs [43, 44, 22] calls for the development of theoretical models that allow one to quantify protein energetics affected/modulated by the binding of allosteric effectors. In this context, an ideal model should be the result of the synthesis between the thermodynamic and structural views of allostery [14]. Such a comprehensive model should provide an allosteric free energy at the single residue level, a key quantity for evaluating the free energy associated with the processes of allosteric regulation. The model should also provide quantification of the free energy difference between sequential steps of binding of identical and/or different types of effector molecules, as well as to distinguish effects of agonists and antagonists on the protein’s functional activity.

The structure-based statistical mechanical model of allostery introduced here consists of three key components: (i) it models the effector binding via local perturbation/stabilization of the protein harmonic model; (ii) per residue allosteric potential estimates structural changes due to the effect of protein dynamics on the residue environment; (iii) statistical mechanical ensemble treatment provides a quantification of the allosteric free energy obtained from the per-residue partition functions in the ligand free and bound states. The model is applied to a large set of allosteric proteins previously analyzed in the context of allosteric site prediction [21]. The list of proteins is heterogeneous in terms of their functions, degree of oligomerization, sizes, and allosteric phenomenologies. We found a variety of modes of allosteric regulation, e.g. binding in allosteric sites causes different levels of tension or relaxation at functional sites. These observations show that allosteric communication between protein sites is a direct consequence of the configurational work redistribution according to structural peculiarities of the allosterically regulated proteins. The calculated changes in stability of catalytic sites, i.e. configurational work exerted at these sites, are found to be strongly dependent on the topology, oligomerization state, and other structural/functional traits. A paradigm case is, for example, D-3-phosphoglycerate dehydrogenase (PGDH) which shows how the redistribution of configurational work strongly resembles the domain structures of the protein and its functional mechanisms. Stability modulation, in turn, appeared to be more dependent on the type of the allosteric regulation: in case of Phosphofructokinase (PFK) it is a result of the interplay between binding of different ligands. In particular, PEP (allosteric inhibitor) and ADP (substrate and/or activator depending on the location) are both responsible for the stability regulation of the F6P binding site and for the positive cooperativity of the F6P binding. In the case of catabolite activator protein (CAP) the sequential scenario of binding to two allosteric cAMP sites delineates the negative cooperativity scenario in allosteric regulation of the DNA binding. Overall, a clear picture with good qualitative agreement between our observations and experimentally described modes of regulation was obtained. Looking forward to the next steps in this work, it is important to briefly discuss the major advantages of the model as well as its drawbacks. The first obvious limitation stems from its coarse-grained nature (*C*_*α*_ harmonic model), since the lack of atomic detail affects the quality of the allosteric effects’ estimation. An indirect modeling of the binding effect can be considered as both a limitation and an advantage of the model. First, it does not take into account the structure of the ligands or the actual set of interactions in the binding sites. On the other hand, the indirect way of mimicking the ligand binding is a generic framework that can be applied to different types of proteins without any preliminary knowledge of the allosteric sites. While the model could benefit from empirical parametrization on the basis of experimental observations, it provides an energy estimate of the allosteric signaling.

To conclude, this approach should be regarded as a *ground state* *model,* as the reference structure used in a harmonic model of a protein is the same in the ligand free and ligand bound systems. The free energy difference between the ligand free and ligand bound states is chiefly determined by the contribution of the configurational entropy. Allosteric communication detected by the model is indicative of the signaling between the regulatory and functional sites in both directions. Therefore, perturbation of the functional site can point to the potential locations of relevant the allosteric ones. As a result, the perturbation of the functional sites would allow one to determine the allosteric sites that modulate activity of corresponding catalytic sites, to identify specifics of the allosteric communication at the atomic level resolution, and to discriminate between the agonism and antagonism of allosteric effectors.

## Materials and Methods

### Harmonic model of free and bound states

A proper choice for the description of protein dynamics is a key component in modeling of the allosteric processes. For the sake of computational simplicity we adopt here a description of the protein configurational states based on a *C*_*α*_ harmonic model. Despite this radical simplification, harmonic models are surprisingly powerful in describing the large-amplitude protein motions based on the consideration of the low-frequency normal modes [45–47]. An effective harmonic potential introduced in [48] is used in this work within the implementation given in MMTK simulation package [49]. The pairwise effective energy function between all *C*_*α*_ atoms is

$$\begin{array}{ccc} E^{\left(0\right)}\left(r-r^{0}\right) & = & \sum_{\text{pairs}\;i,j}k_{ij}{\left(d_{ij}-d_{ij}^{0}\right)}^{2} \end{array}$$ (7)

where **r** is the 3N-dimensional vector of coordinates of the *C*_*α*_ atoms, **r**^0^ is a vector of *C*_*α*_ positions of the reference structure, *d*_*ij*_ is the Euclidian distance between the *C*_*α*_ atoms *i* and *j*, $d_{ij}^{0}$ is the corresponding distance in the reference structure, and *k*_*ij*_ is the distance-dependent force constant decaying as ${\left(1/d_{ij}^{0}\right)}^{6}$ with a global 25 Å distance cutoff (see default settings in [48]). In order to estimate effects of the binding to different sites (or their combinations) on the protein energetics, we consider two harmonic systems: the free system P with energy given by eq (7) and the system $A^{\left(n\right)}P$ with *n* ligands bound to sites *s*_1_, …, *s*_*n*_. We define the latter by adding a binding site’s stiffening harmonic term to the energy function of the free system, that is

$$E^{\left(n\right)}\left(r-r^{0}\right)=E^{\left(0\right)}\left(r-r^{0}\right)+\alpha \sum_{k}V_{s_{k}}\left(r-r^{0}\right)$$ (8)

with the additional terms $V_{s}\left(r-r^{0}\right)=1/2\sum_{\text{pairs}\;i,j\in s}k_{ij}{\left(d_{ij}-d_{ij}^{0}\right)}^{2}$ corresponding to the restrained binding site *s* and *α* > 0 is the stiffening factor. The effect of ligand binding is indirectly modeled as a stabilization effect on the atoms that belong to the binding site of interest. Thus, the factor *α* gives the extent to which these indirect interactions affect the atoms of the binding site. To ensure a pronounced allosteric effect, a stiffening factor *α* = 100 was chosen based on the analysis of the allosteric free energy profiles obtained for the list of proteins (see S1 Fig). From the Hessian matrices **K**^(0)^ = ∂^2^ *E*^(0)^/∂**r**_*i*_∂**r**_*j*_ and **K**^(*n*)^ = ∂^2^ *E*^(*n*)^/∂**r**_*i*_∂**r**_*j*_ the orthonormal normal modes $e_{\mu }^{\left(0\right)}$, $e_{\mu }^{\left(n\right)}$ were calculated for the ligand free and ligand bound systems P, $A^{\left(n\right)}P$, respectively.

The additional harmonic terms in eq 8 induce a reorganization of the normal modes of the free system, consistent with the observation that ligand binding causes an overall change in the protein dynamics by shifting from low to high values in the normal mode frequency spectrum [12, 50, 51]. Formulated in energy terms, restraint of the degrees of freedom in a binding site essentially mimics an additional energy that is supplied to the system in the case of the actual ligand binding. This energy supply can be “communicated” to other functionally relevant protein regions, and it can be expressed in terms of the gain/loss of the work exerted in corresponding parts of the protein. Similar *perturbation-based* harmonic models have been employed in previous works [52–55] with a special emphasis on the role of dynamics of individual residues in relation to allosteric binding and mutations. Fig 1A contains a pictorial illustration of the harmonic protein model used in this work for defining the ligand free and ligand bound protein systems. In many cases, there can be several effector and substrate ligands that can bind to a protein independently or in different combinations. In order to give a generalized description of the above cases, we switch here from the binary terminology of the apo (free) and holo (bound) forms of the protein to the notations free and ligated forms of the protein (or binding site).

#### Allosteric potential

In the previous section the sets of normal modes were obtained for the ligand free and bound protein states. Here, the normal modes are used as a basis for defining an allosteric potential in the context of a statistical mechanical framework. We are interested in evaluating the effect of the ligand binding on an arbitrary chosen residue *i* of the protein. In order to do that, we introduce an allosteric potential of residue *i* determined by the normal mode *μ* as:

$$\begin{array}{ccc} U_{\mu ,i} & = & \frac{1}{2}\sum_{j:d_{ij}^{0}<d_{c}}c{\left(d_{ij}^{\left(\mu \right)}-d_{ij}^{0}\right)}^{2} \end{array}$$ (9)

with *c* = 1 kcal/mol/Å^2^, *d_c_* = 11Å a distance cutoff, $d_{ij}^{0}$ are *C*_*α*_ distances in the reference structure *R*, and $d_{ij}^{\left(\mu \right)}$ are *C*_*α*_ distances as a result of the normal mode *μ*. Thus, the potential evaluates the total elastic work that is exerted on the residue *i* as a result of the deforming action of a normal mode *μ* on its neighboring residues *j*. The idea behind the allosteric potential per residue *i* and mode *μ* is to compare the different deforming effects on a residue *i* induced by the normal mode of the free system ($e_{\mu }^{\left(0\right)}$) and of the system with *n* bound ligands ($e_{\mu }^{\left(n\right)}$), respectively. Fig 1B illustrates how the neighbors of an residue *i* (residues *j*, *k*, *l*) undergo different displacements upon a normal mode *μ*, changing the inter-residue distances $d_{ij}^{\left(0,\mu \right)}$ and $d_{ij}^{\left(n,\mu \right)}$ in the free and ligated sites.

The displacement of the residue *i* due to mode **e**_*μ*_ is $r_{i}^{\left(\mu \right)}-r_{i}^{0}=\sigma_{\mu }e_{\mu ,i}$ where $r_{i}^{0}$ are the coordinates of residue *i* and σ_μ_ is a gaussian coefficient. Thus, the allosteric potential in eq (9) can be rewritten as (to avoid redundancy we focus on the *n* ligated binding sites only)

$$U_{\mu ,i}^{\left(n\right)}\left(\sigma_{\mu }\right)=\frac{1}{2}\varepsilon_{\mu ,i}^{\left(n\right)}\sigma_{\mu }^{2}$$ (10)

in terms of the coefficients *σ*_*μ*_ and residue intensity parameters

$$\varepsilon_{\mu ,i}^{\left(n\right)}=\sum_{j:d_{ij}^{0}<d_{c}}c{\left|e_{\mu ,i}^{\left(n\right)}-e_{\mu ,j}^{\left(n\right)}\right|}^{2}$$ (11)

Summing over the modes of interest, the total allosteric potential of the residue *i* is

$$U_{i}^{\left(n\right)}\left(\sigma \right)=\frac{1}{2}\sum_{\mu }\varepsilon_{\mu ,i}^{\left(n\right)}\sigma_{\mu }^{2}$$ (12)

where *σ* = (*σ*_1_, *σ*_2_, …) is a set of gaussian coefficients that can be regarded as a *configurational state* of a residue *i*.

#### Free energy of ligand binding and allosteric communication

In a statistical thermodynamic fashion the configurational free energy of a harmonic model is simply given by the expression *G* = 1/2*k*_*B*_ *T*∑_*μ*_ ln*λ*_*μ*_ + *const*, with *λ*_*μ*_ the eigenvalues of the Hessian matrix **K** of the model ($\lambda_{\mu }=\omega_{\mu }^{2}$ with *ω*_*μ*_ the frequency associated to the mode *μ*). Thus, the free energy difference between the state with *n* bound ligands $A^{\left(n\right)}P$ and the ligand free state P is:

$$\Delta G\left(P\to A^{\left(n\right)}P\right)=\frac{1}{2}k_{B}T\sum_{\mu }\ln\frac{\lambda_{\mu }^{\left(n\right)}}{\lambda_{\mu }^{\left(0\right)}}$$ (13)

and the modulating free energy

$$\Delta \Delta G\left(P\to A^{\left(m\right)}P\to A^{\left(n\right)}P\right)=\frac{1}{2}k_{B}T\sum_{\mu }\ln\frac{\lambda_{\mu }^{\left(0\right)}\lambda_{\mu }^{\left(n\right)}}{{\left(\lambda_{\mu }^{\left(m\right)}\right)}^{2}}$$ (14)

where an intermediate case with *m* ligands bound $A^{\left(m\right)}P$ state is defined by the *m* < *n*. The relations in Eqs (13) and (14) are global parameters, which quantify the configurational work (allosteric free energy) gain/loss of the structure’s conformational ensemble that is exerted by the protein as a consequence of having multiple binding sites occupied [51].

Contrary to the above generic approach, our goal here is to quantify the allosteric effects at the single residue level, estimating the allosteric free energy per residue. To this end, the introduced allosteric potential eq (12) is now used for obtaining the canonical partition functions $z_{i}^{\left(0\right)},z_{i}^{\left(n\right)}$ of residue *i* in the ligand free and *n*-ligated states of the protein, respectively. The set of coefficients *σ* = (*σ*_1_, *σ*_2_…) in eq (10) identifies an atomic configuration that corresponds to a particular linear combination of the low frequency normal modes. The coefficients *σ*_*μ*_ are gaussian variables with zero mean and variance 1/*ε*_*μ*, *i*_ (eq 11). For example, the per residue partition function for the state with *n* bound ligands, $z_{i}^{\left(n\right)}$, that corresponds to the ensemble of all possible configurations is

$$\begin{array}{ccc} z_{i}^{\left(n\right)} & = & \int d\sigma e^{-U_{i}^{\left(n\right)}\left(\sigma \right)/k_{B}T}\\ & = & \prod_{\mu }\int d\sigma_{\mu }e^{-\sigma_{\mu }^{2}\varepsilon_{\mu ,i}^{\left(n\right)}/2k_{B}T}\\ & = & \prod_{\mu }{\left(\pi \frac{2k_{B}T}{\varepsilon_{\mu ,i}^{\left(n\right)}}\right)}^{1/2} \end{array}$$ (15)

The free energy per residue is *g*_*i*_ = −*k*_*B*_ *T* ln*z*_*i*_ + *const* so that the allosteric free energy difference per residue between the *n*-ligated state $A^{\left(n\right)}P$ and the free state P is

$$\Delta g_{i}\left(P\to A^{\left(n\right)}P\right)=\frac{1}{2}k_{B}T\sum_{\mu }\ln\frac{\varepsilon_{\mu ,i}^{\left(n\right)}}{\varepsilon_{\mu ,i}^{\left(0\right)}}$$ (16)

and the corresponding modulating free energy is

$$\Delta \Delta g_{i}\left(P\to A^{\left(m\right)}P\to A^{\left(n\right)}P\right)=\frac{1}{2}k_{B}T\sum_{\mu }\ln\frac{\varepsilon_{\mu ,i}^{\left(n\right)}\varepsilon_{\mu ,i}^{\left(0\right)}}{{\left(\varepsilon_{\mu ,i}^{\left(m\right)}\right)}^{2}}$$ (17)

with the intermediate *m*-ligated $A^{\left(m\right)}P$ state *m* < *n*. The free energies in eqs (16) and (17) quantify the maximal configurational work $\Delta g_{i}\left(P\to A^{\left(n\right)}P\right)$ that is exerted on a residue *i* as a consequence of the change in the ensemble of configurations assumed by the residue’s neighbors as a result of ligand binding. It is clear that the free energy in eq (16) represents the change in stability of a residue *i* caused by the allosteric signaling. Similarly, the expression in eq (17) evaluates the modulating effect on the stability (configurational work) of a residue *i* caused by changing the number of ligated allosteric sites. In Fig 1C the values of the free energies per residue, are represented via the thickness of the protein backbone (a tube-like representation is used).

The obtained free energies in the eqs (16) and (17) clearly depend on the number of low frequency normal modes. The dependence of the free energy profiles per residue on the number of normal modes (we used 5, 10, 25, and 50 modes) was analyzed for a subset of proteins (CAP, BGDH, and PFK). The results of these calculations are shown in the S2 Fig. There is no strong qualitative difference between the free energy profiles obtained with 10 and 50 modes. We concluded, therefore, that ten normal modes can already provide qualitatively correct picture, which can be further refined by the inclusion of additional modes. The choice of using the first ten low frequency modes is also corroborated by the previous studies on the same set of proteins [21, 15], where it was shown that the first ten normal modes fairly well described conformational transitions and allosteric communication.

#### Protein data set and computational framework

The method introduced in this work is applied to the list of allosteric enzymes used in previous studies in the context of binding leverage and leverage coupling calculations [15, 21]. Most of the proteins in the list (ATCase, AnthS, BGDH, CAP, DAHPS, DAK, G6PD, NADME, PFK, PGDH, PKA, PTP1B, SSUPRT, ThrS) are homo-oligomeric complexes, except two hetero-oligomers (ATCase, AnthS) and two monomeric forms (PKA, PTP1B). For each of the analyzed proteins at least two forms were used (apo form whenever that was available). All the calculations considered the structures cleaned of the all bound ligands. The actual protein assemblies (oligomeric structures) used as an input in this work were obtained from the PDBePISA (Proteins, Interfaces, Structures and Assemblies) interactive tool [56].

The protein modeling as well as the normal mode calculations were carried out with the MMTK package [49]. The *C*_*α*_ effective harmonic model described in [48] was used to characterize the dynamics of the ligand free and ligand bound protein systems. Specifically, the combination of the Harmonic Distance Restraint module with the Compound Force Field feature were used to construct the harmonic model associated to the ligand bound protein. The Fourier approximation was used for the calculations of the normal modes [57]. The MMTK calculations as well as the implementation of the three steps of the model presented above were coded in an iPython notebook which is available upon request.

## Supporting Information

S1 Fig The plots showing the dependence of the allosteric free energy profiles on the different values of the stiffening parameter *α* that characterize the ligand bound protein states. (PDF)

S2 Fig The plots showing the dependence of the allosteric free energy profiles on the number of used normal modes. (PDF)

S3 Fig The allosteric free energy profiles of PKA and PTP1B proteins. (PDF)

S1 Table Results on allosteric causality and energetics obtained for proteins analyzed in this work. The table includes the complete data obtained for all available forms of proteins. For designation of columns see Table 1 in the main text.
(PDF)
