# Supplementary material for: Structure-Based Statistical Mechanical Model Accounts for the Causality and Energetics of Allosteric Communication
Source: PLoS Comput Biol. 2016 Mar 3;12(3):e1004678. doi: 10.1371/journal.pcbi.1004678 (PMC4777440; doi:10.1371/journal.pcbi.1004678)
Supplement: S1 Fig — (PDF) [file pcbi.1004678.s001.pdf]

S1 Figure

The dependence of the allosteric free energy profiles on increasing values of the stiffening parameters α.

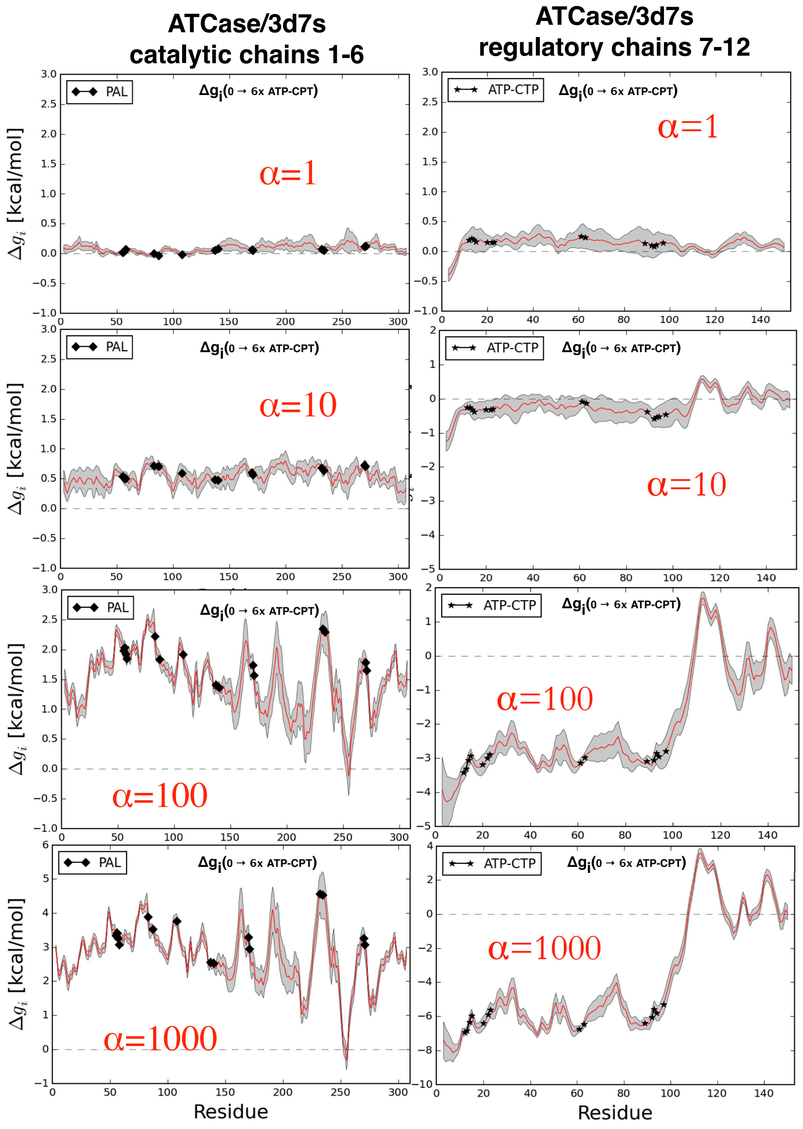

Aspartate carbamoyltransferase (ATCase)

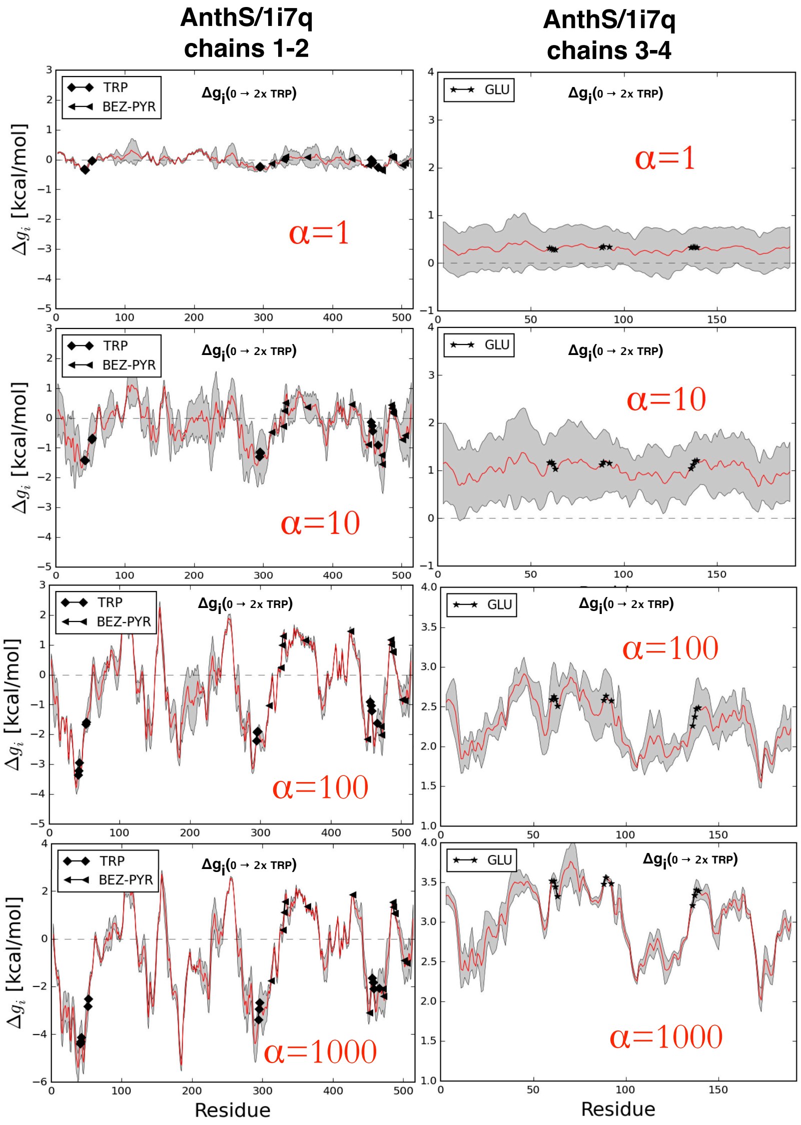

Anthranilate Synthase (AnthS)

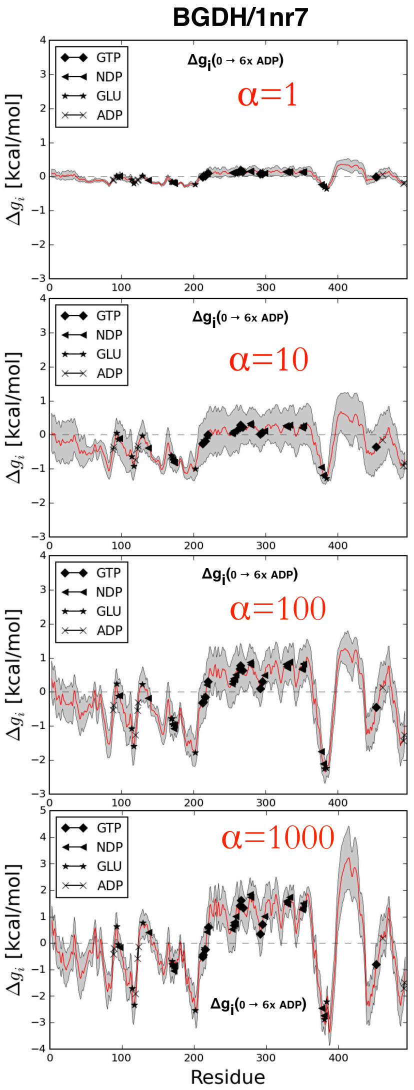

Bovine glutamate dehydrogenase (BGDH)

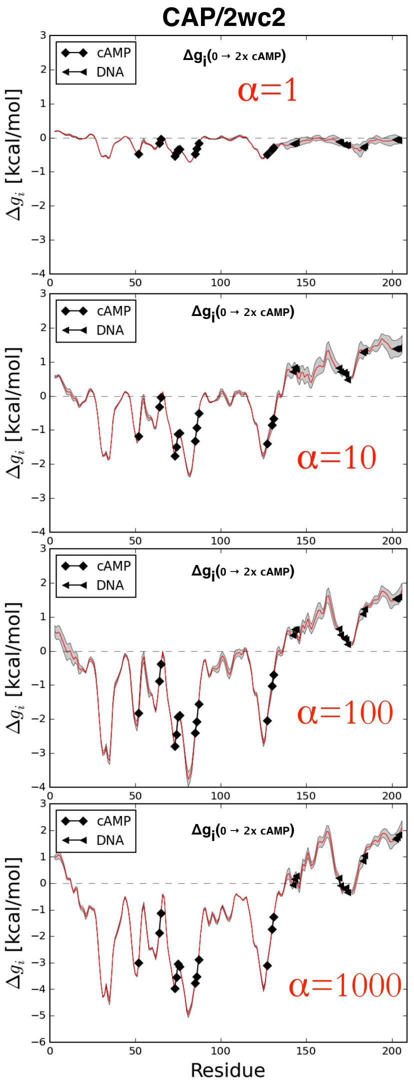

Catabolite Activator Protein (CAP)

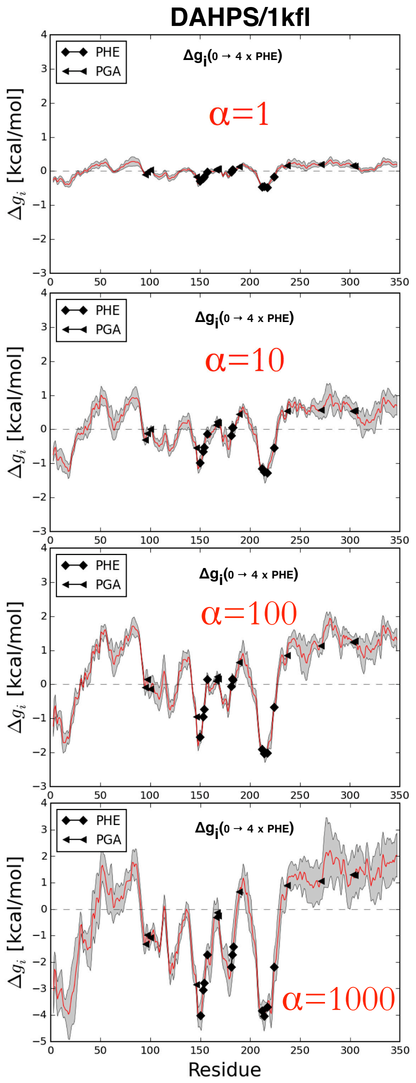

3-deoxy-D-arabinoheptulosonate 7-phosphate synthase (DAHPS)

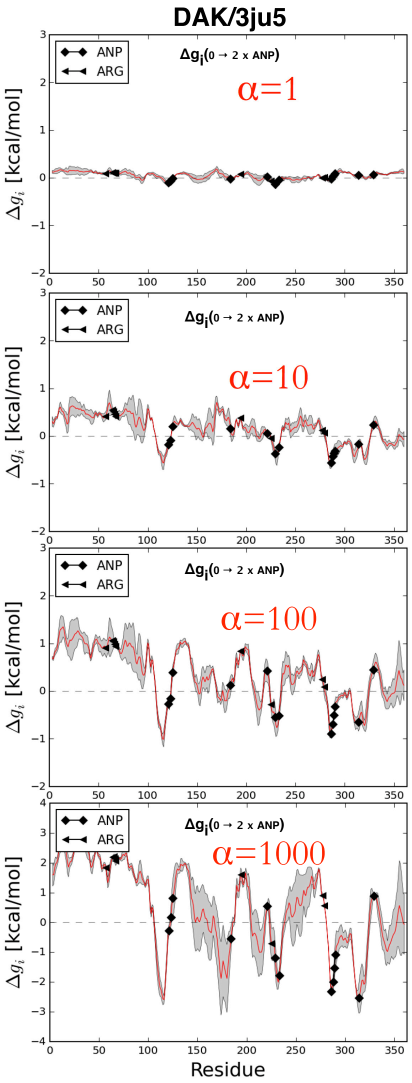

Dihydroxyacetone kinase (DAK)

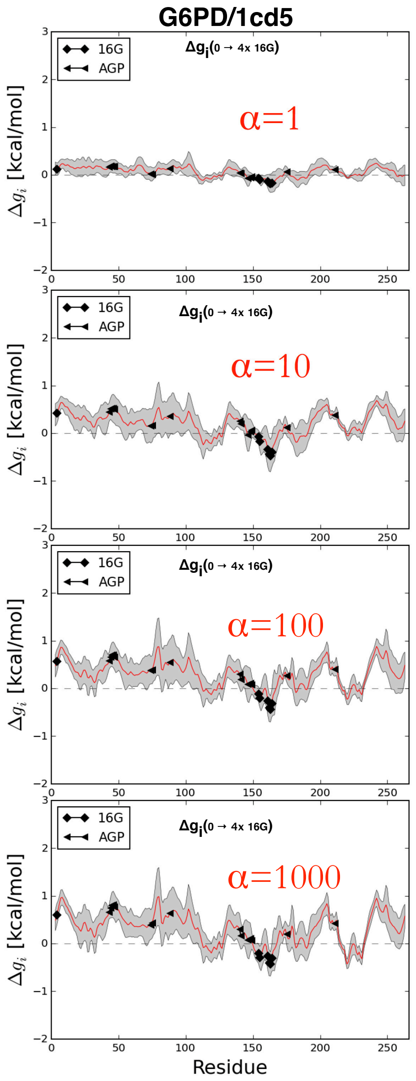

Glucose-6-phosphate dehydrogenase (G6PD)

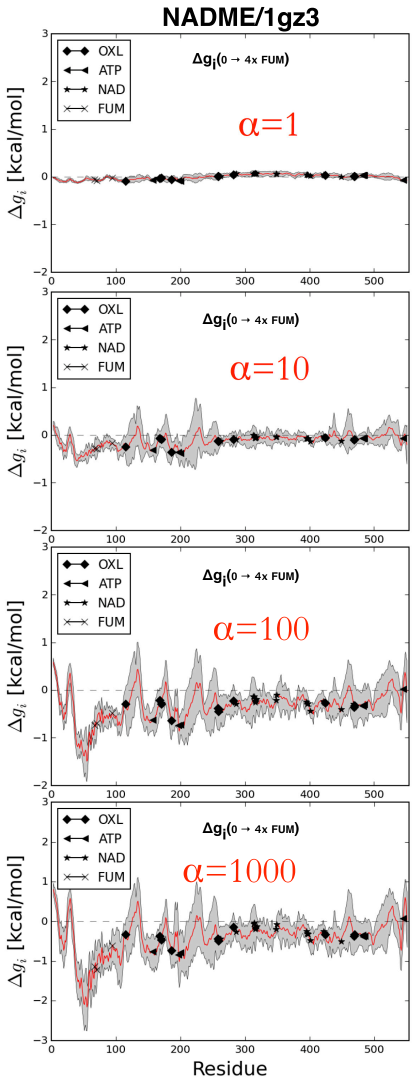

Malate dehydrogenase (decarboxylating) enzyme (NADME)

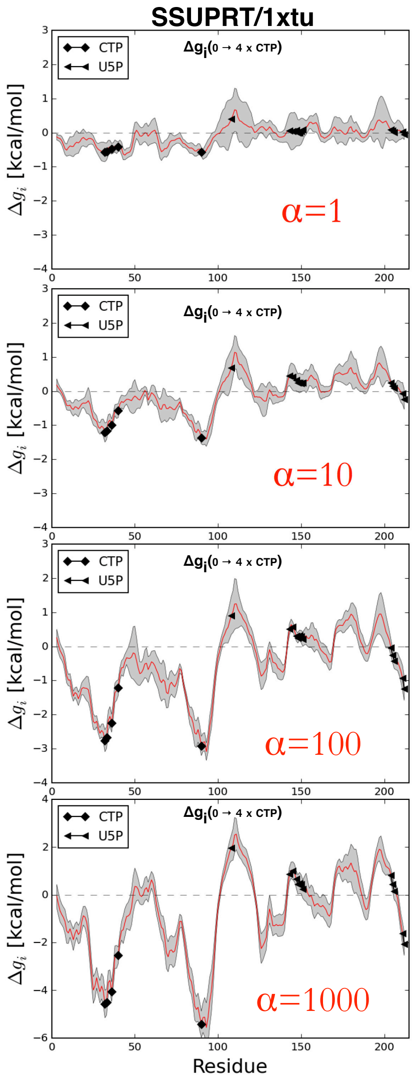

Sulfolobus solfataricus uracil phosphoribosyltransferase (SSUPTR)

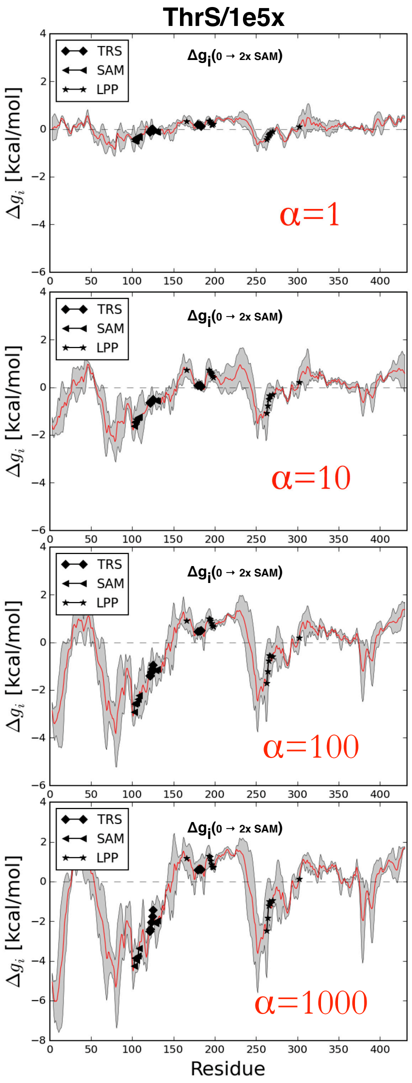

Threonine synthase (ThrS)

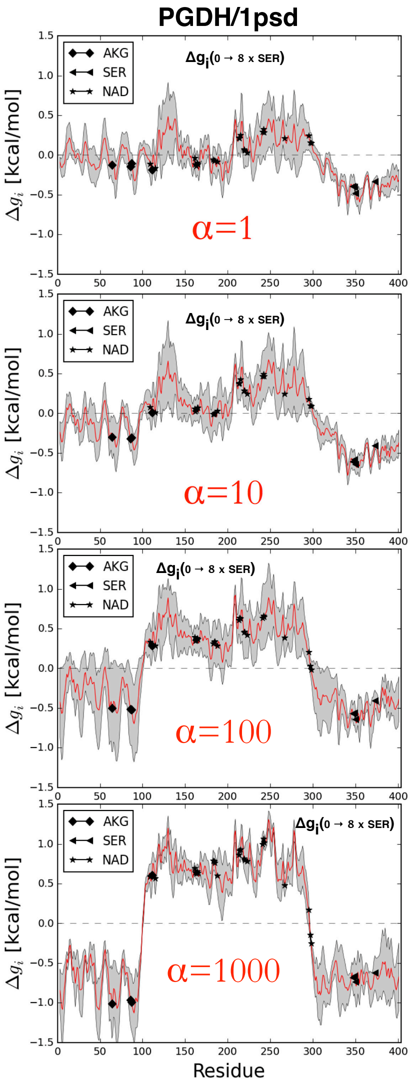

D-3-phosphoglycerate dehydrogenase (PGDH)

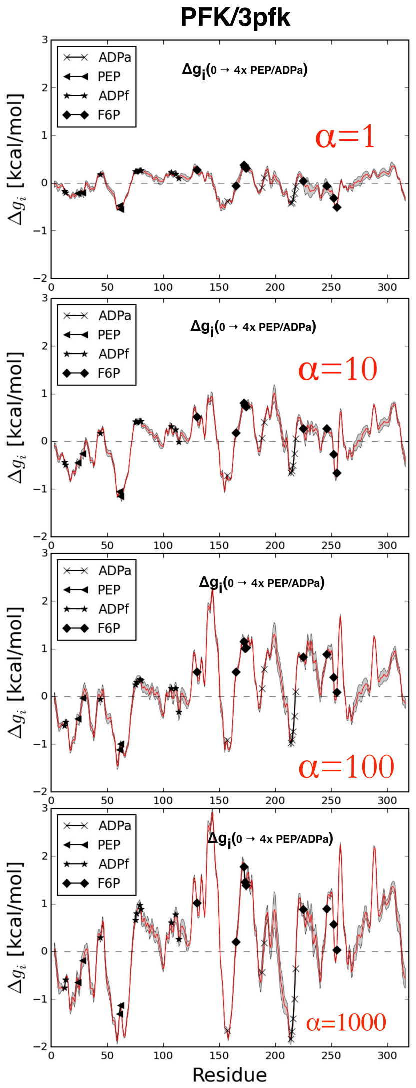

Phosphofructokinase (PFK)
