# Supplementary material for: Structure-Based Statistical Mechanical Model Accounts for the Causality and Energetics of Allosteric Communication
Source: PLoS Comput Biol. 2016 Mar 3;12(3):e1004678. doi: 10.1371/journal.pcbi.1004678 (PMC4777440; doi:10.1371/journal.pcbi.1004678)
Supplement: S2 Fig — (PDF) [file pcbi.1004678.s002.pdf]

S2 Figure

The dependence of the allosteric free energy profiles for increasing size of the set of low frequency normal modes.

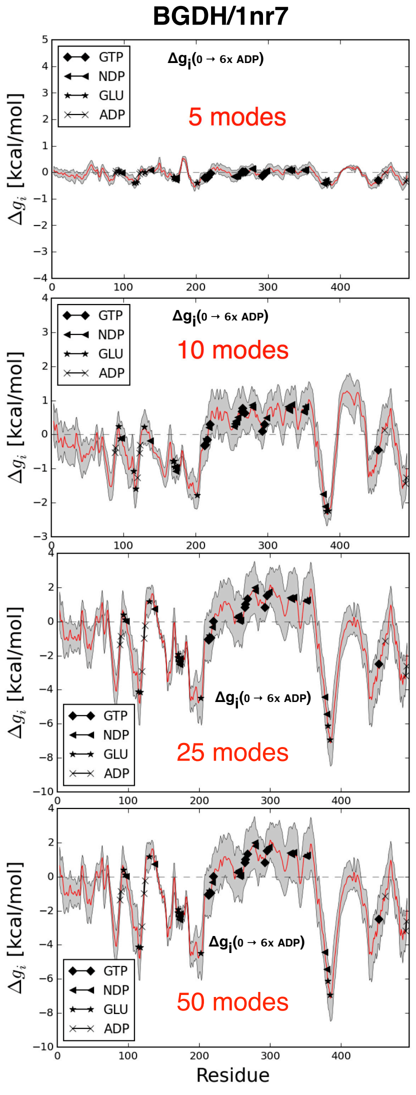

Bovine glutamate dehydrogenase (BGDH)

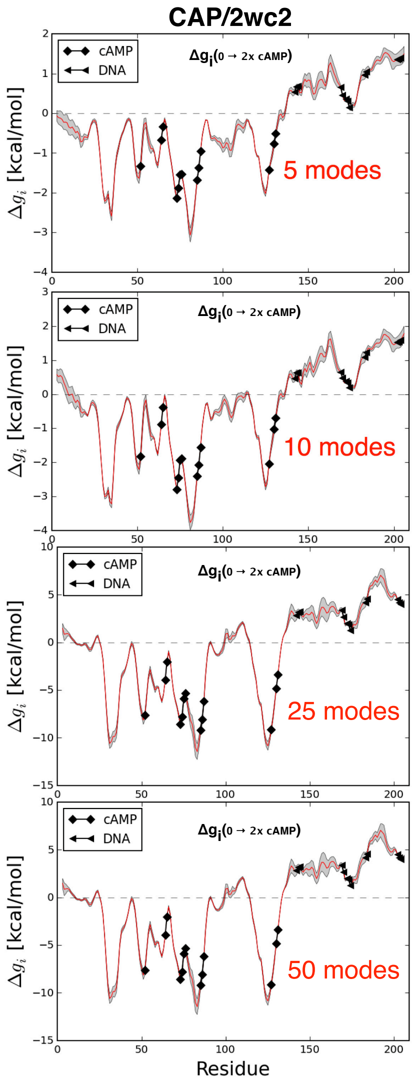

Catabolite Activator Protein (CAP)

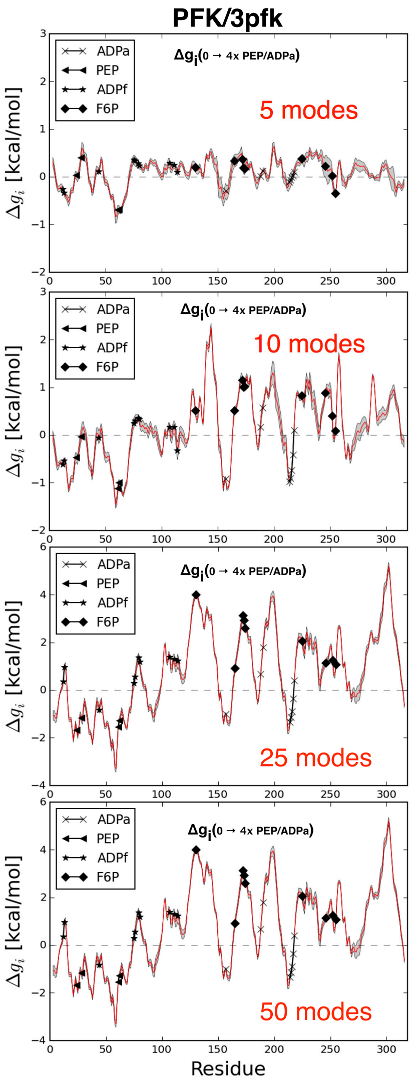

Phosphofructokinase (PFK)
