# Supplementary material for: Structure-Based Statistical Mechanical Model Accounts for the Causality and Energetics of Allosteric Communication
Source: PLoS Comput Biol. 2016 Mar 3;12(3):e1004678. doi: 10.1371/journal.pcbi.1004678 (PMC4777440; doi:10.1371/journal.pcbi.1004678)
Supplement: S3 Fig — (PDF) [file pcbi.1004678.s003.pdf]

S3 Figure

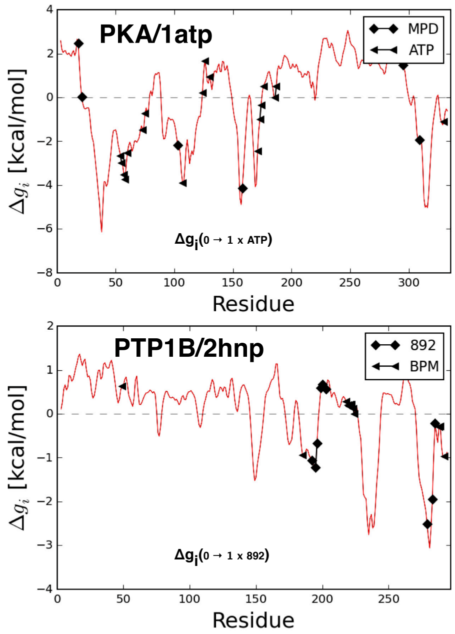

The allosteric free energy profiles Δg_i for the proteins DAK (apo form PDB ID: 1atp) and PTP1B (apo form PDB ID: 2hnp) as a result of the allosteric binding of at the ATP and 892 sites respectively.
