# Supplementary material for: Structure-Based Statistical Mechanical Model Accounts for the Causality and Energetics of Allosteric Communication
Source: PLoS Comput Biol. 2016 Mar 3;12(3):e1004678. doi: 10.1371/journal.pcbi.1004678 (PMC4777440; doi:10.1371/journal.pcbi.1004678)
Supplement: S1 Table — The table includes the complete data obtained for all available forms of proteins. For designation of columns see Table 1 in the main text. (PDF) [file pcbi.1004678.s004.pdf]

S1 Table

Protein<br>(#ch, #res)	PDB ID<br>(ligands)	Ligated<br>Site (A/I/S)	$\Delta g_A(\mathcal{P} \to A\mathcal{P})$ $(\Delta g_{mer}(\mathcal{P} \to A\mathcal{P}))$<br>kcal/mol	Regulated<br>Site	$\Delta g_F(\mathcal{P} \to A\mathcal{P})$ $(\Delta g_{mer}(\mathcal{P} \to A\mathcal{P})$<br>kcal/mol
ATCase<br>(2x3+3x2, 2736)	3d7s<br>(apo)	6 x ATP/CTP (A/I)	-3.35 (-2.02)$^{(*)}$	PAL	1.98 (1.47)$^{(**)}$
	1rac<br>(CTP)	6 x ATP/CTP (A/I)	-3.27 (-1.61)$^{(*)}$	PAL	1.94 (1.83)$^{(**)}$
	7ati<br>(PAL, ATP)	6 x ATP/CTP (A/I)	-2.80 (-1.55)$^{(*)}$	PAL	1.88 (1.73)$^{(**)}$
	1d09<br>(PAL)	6 x ATP/CTP (A/I)	-2.32 (-1.35)$^{(*)}$	PAL	1.28 (1.18)$^{(**)}$
AnthS<br>(2+2, 1426)	1i7q<br>(BEZ, PYR, GLU)	2 x TRP (I)	-2.87 (-0.42)$^{(*)}$	BEZ+PYR<br>GLU	-0.60 (-0.42)$^{(*)}$<br>2.13 (2.29)$^{(**)}$
	1i7s<br>(TRP)	2 x TRP (I)	-2.14 (-0.28)$^{(*)}$	BEZ+PYR<br>GLU	-0.11 (-0.28)$^{(*)}$<br>1.63 (1.90)$^{(**)}$
BGDH<br>(6, 2976)	1nr7<br>(apo)	6 x ADP (A)	-1.51 (-0.16)	NDP<br>GLU	0.11<br>-0.88 (-0.16)
		6 x GTP (I)	-0.32 (0.61)	NDP<br>GLU	0.14<br>0.47 (0.61)
		6 x ADP (A)<br>GTP (I)	-0.64<br>-0.40 (0.60)	NDP<br>GLU	0.32<br>-0.01 (0.60)
	1hwz<br>(NDP,GLU, GTP)	6 x ADP (A)	-1.41 (-0.41)	NDP<br>GLU	-0.55<br>-0.83 (-0.41)
		6 x GTP (I)	-1.61 (-0.11)	NDP<br>GLU	-0.94<br>-0.19 (-0.11)
		6 x ADP (A)<br>GTP (I)	-1.06<br>-2.18 (-0.32)	NDP<br>GLU	-1.18<br>-0.62 (-0.32)
	1nqt<br>(ADP)	6 x ADP (A)	-1.32 (0.02)	NDP<br>GLU	0.26<br>-0.66 (0.02)
		6 x GTP (I)	-0.26 (0.65)	NDP<br>GLU	0.23<br>0.49 (0.65)
		6 x ADP (A)<br>GTP (I)	-0.52<br>-0.31 (0.77)	NDP<br>GLU	0.52<br>0.16 (0.77)
CAP<br>(2, 418)	2wc2	1 x cAMP (A)	-1.49 (-0.13)$^{(\sharp)}$	DNA<br>cAMP	0.88<br>-1.06 (0.24)$^{(\sharp\sharp)}$
	(apo)	2 x cAMP (A)	-2.37 (-0.30)	DNA	0.85 (-0.30)
	1run	1 x cAMP (A)	-2.07 (-0.64)$^{(\sharp)}$	DNA<br>cAMP	-0.41<br>-0.55 (-0.08)$^{(\sharp\sharp)}$
	(cAMP)	2 x cAMP (A)	-3.32 (-0.49)	DNA	0.44 (-0.49)
	1o3q	1 x cAMP (A)	-1.80 (-0.47)$^{(\sharp)}$	DNA<br>cAMP	-0.09<br>0.10 (0.67)$^{(\sharp\sharp)}$
	(cAMP,DNA)	2 x cAMP (A)	-1.53 (-0.52)	DNA	-0.26 (-0.52)
DAHPS<br>(4, 1401)	1gg1<br>(PGA)	4 x PHE (I)	-2.33 (0.25)	PGA	0.33 (0.25)
	1kfl<br>(PGA, PHE)	4 x PHE (I)	-1.36 (0.40)	PGA	0.31 (0.40)
DAK<br>(2, 735)	3ju5<br>(apo)	1 x ADP (S)<br>ARG (S)	-1.93<br>-0.76 (-0.72)	ADP<br>ARG	0.49<br>1.03 (0.74)
		2 x ADP (S)<br>ARG (S)	-0.85<br>-0.11 (-0.05)	ADP<br>ARG	-0.85<br>-0.11 (-0.05)
	3ju6<br>(ANP, ARG)	1 x ADP (S)<br>ARG (S)	-1.49<br>-0.69 (-0.60)	ADP<br>ARG	1.32<br>0.95 (1.01)
		2 x ADP (S)<br>ARG (S)	-0.91<br>-0.33 (0.24)	ADP<br>ARG	-0.91<br>-0.33 (0.24)

S1 TABLE. Results on allosteric causality and energetics obtained for proteins analyzed in this work. Complete data obtained for all available forms of proteins. For designation of columns see Table 1 in the main text.

Protein (#ch, #res)	PDB ID (ligands)	Ligated Site (A/I/S)	$\Delta g_A(\mathcal{P} \rightarrow A\mathcal{P})$ ($\Delta g_{mer}(\mathcal{P} \rightarrow A\mathcal{P})$) kcal/mol	Regulated Site	$\Delta g_F(\mathcal{P} \rightarrow A\mathcal{P})$ ($\Delta g_{mer}(\mathcal{P} \rightarrow A\mathcal{P})$) kcal/mol
G6PD (6, 1596)	1cd5 (apo)	6 x 16G (A)	-0.04 (0.31)	AGP	0.37 (0.31)
	1hor (AGP)	6 x 16G (A)	-0.37 (0.17)	AGP	0.25 (0.17)
	1hot (16G)	6 x 16G (A)	-0.36 (0.10)	AGP	0.16 (0.10)
NADME (4, 2232)	1gz3 (FUM, ATP)	4 x FUM (A)<br>4 x ATP (S/I)	-0.85 (-0.32)<br>-1.23 (0.19)	NAD<br>NAD	-0.40 (-0.32)<br>0.78 (0.19)
	1efk (NAD)	4 x FUM (A)<br>4 x ATP (S/I)	-0.53 (-0.14)<br>-1.42 (-0.25)	NAD<br>NAD	-0.14 (-0.14)<br>0.11 (-0.25)
PFK (4, 1284)	3pfk (apo)	4 x PEP/ADPa (I/A)	-0.78 (0.16)	F6P<br>ADPf	0.71<br>-0.11 (0.16)
		4 x ADPf (S/A)	-0.74 (0.50)	F6P	1.74 (0.50)
		4 x PEP/ADPa (I/A)<br>ADPf (S/A)	0.35<br>-1.08 (0.68)	F6P	2.22 (0.68)
	4pfk (F6P, ADP)	4 x PEP/ADPa (I/A)	-0.71 (0.11)	F6P<br>ADPf	0.76<br>0.19 (0.11)
		4 x ADPf (S/A)	-0.79 (0.40)	F6P	1.64 (0.40)
		4 x PEP/ADPa (I/A)<br>ADPf (S/A)	-0.10<br>-0.74 (0.55)	F6P	2.06 (0.55)
	6pfk (PEP)	4 x PEP/ADPa (I/A)	-1.55 (0.08)	F6P<br>ADPf	0.64<br>0.60 (0.08)
		4 x ADPf (S/A)	-0.64 (0.64)	F6P	1.78 (0.64)
		4 x PEP/ADPa (I/A)<br>ADPf (S/A)	-0.34<br>-0.78 (0.71)	F6P	2.39 (0.71)
PGDH (4, 1624)	1psd (SER ,NAD)	8 x SER (I)	-0.58 (0.03)	AKG<br>NAD	0.08<br>0.38 (0.03)
	1yba (AKG, NAD)	8 x SER (I)	0.23 (0.09)	AKG<br>NAD	0.05<br>0.33 (0.09)
PKA (1, 336)	1atp (ATP)	1 xATP (I)	-4.28 (-0.10)	MPD	1.97 (-0.10)
	1j3h (MPD)	1 xATP (I)	-3.54 (0.18)	MPD	1.55 (0.18)
PTP1B (1, 278)	2hnp (apo)	1 x 892 (I)	-3.63 (-0.05)	BPM	0.36 (-0.05)
	1aax (BPM)	1 x 892 (I)	-1.37 (0.07)	BPM	0.30 (0.07)
	1t49 (892)	1 x 892 (I)	-3.52 (-0.20)	BPM	0.55 (-0.20)
SSUPRT (4, 868)	1xtu (U5P, CTP)	4 x CTP (I)	-2.89 (-0.53)	U5P	0.14 (-0.53)
	1xtt (U5P)	4 x CTP (I)	-2.62 (-0.01)	U5P	0.65 (-0.01)
ThrS (2, 884)	1e5x (apo)	1 x SAM (A)	-1.31 (0.02)	TRS<br>PLP	-0.31<br>0.35 (0.02)
		2 x SAM (A)	-3.67 (-0.24)	TRS<br>PLP	-0.54<br>0.52 (-0.24)
	2c2g (PLP)	1 x SAM (A)	-2.17 (0.01)	TRS<br>PLP	-0.15<br>0.15 (0.01)
		2 x SAM (A)	-4.48 (-0.20)	TRS<br>PLP	-0.45<br>0.09 (-0.20)
	2c2b (TRS, SAM, PLP)	1 x SAM (A)	-1.89 (-0.67)	TRS<br>PLP	-0.50<br>-0.42 (-0.67)
		2 x SAM (A)	-2.69 (-0.41)	TRS<br>PLP	-1.02<br>0.01 (-0.41)

S1 TABLE (continued)
